# Automated Method for the Rapid and Precise Estimation of Adherent Cell Culture Characteristics from Phase Contrast Microscopy Images

**DOI:** 10.1002/bit.25115

**Published:** 2013-10-05

**Authors:** Nicolas Jaccard, Lewis D Griffin, Ana Keser, Rhys J Macown, Alexandre Super, Farlan S Veraitch, Nicolas Szita

**Affiliations:** 1 Department of Biochemical Engineering, University College LondonTorrington Place, London, WC1E 7JE, United Kingdom; 2 Centre for Mathematics and Physics in the Life Sciences and Experimental Biology, University College LondonLondon, United Kingdom; 3 Department of Computer Science, University College LondonLondon, United Kingdom

**Keywords:** confluency, morphology, cell density, adherent cells, phase contrast microscopy, image-processing, on-line monitoring

## Abstract

The quantitative determination of key adherent cell culture characteristics such as confluency, morphology, and cell density is necessary for the evaluation of experimental outcomes and to provide a suitable basis for the establishment of robust cell culture protocols. Automated processing of images acquired using phase contrast microscopy (PCM), an imaging modality widely used for the visual inspection of adherent cell cultures, could enable the non-invasive determination of these characteristics. We present an image-processing approach that accurately detects cellular objects in PCM images through a combination of local contrast thresholding and *post hoc* correction of halo artifacts. The method was thoroughly validated using a variety of cell lines, microscope models and imaging conditions, demonstrating consistently high segmentation performance in all cases and very short processing times (<1 s per 1,208 × 960 pixels image). Based on the high segmentation performance, it was possible to precisely determine culture confluency, cell density, and the morphology of cellular objects, demonstrating the wide applicability of our algorithm for typical microscopy image processing pipelines. Furthermore, PCM image segmentation was used to facilitate the interpretation and analysis of fluorescence microscopy data, enabling the determination of temporal and spatial expression patterns of a fluorescent reporter. We created a software toolbox (PHANTAST) that bundles all the algorithms and provides an easy to use graphical user interface. Source-code for MATLAB and ImageJ is freely available under a permissive open-source license. Biotechnol. Bioeng. 2014;111: 504–517. © 2013 Wiley Periodicals, Inc.

## Introduction

Development of robust cell culture protocols relies on the ability to accurately assess characteristics of adherent cell populations such as cell number and phenotype. This information is required both to ensure consistency during routine maintenance, and to assess the outcome of experimental investigations. Typical assays, such as cell enumeration using a counting chamber or flow cytometry, require detachment of the cells and are thus disruptive to key characteristics of the cell populations, such as spatial distribution or morphology, preventing collection of this potentially valuable information. Furthermore, as a consequence of their disruptive nature they preclude the collection of time-course data from a single adherent cell population, which in turn constrains the detection of short-term transient or dynamic cellular responses. A non-invasive, analytical method for rapid and precise determination as well as continual monitoring of adherent cultures characteristics would thus clearly benefit areas such as stem cell bioprocessing (Giri and Bader, 2013) and drug discovery (Kepp et al., 2011).

The standard method to assess the visible properties of adherent cultures is inspection by light microscopy. A typical outcome of these inspections is an estimate of confluency, a measure of the fraction of the growth area covered by cells. As a metric, confluency is particularly useful when detachment is not possible, for example to determine when to passage cells (Kato et al., 2010) or when to induce a perturbation (Stewart and Rotwein, 1996; Van den Eijnde et al., 2001). Confluency also informs on the spatial crowding of the cells, a property relatable to *in vivo* cellular tissues, which was shown to have an impact on gene expression (Ruutu et al., 2004), formation of cell–cell junctions (Lampugnani et al., 1997) and the development potential of embryonic stem cells into viable embryos (Gao et al., 2003). Cell morphology is an equally important characteristic of adherent cell cultures. Indeed, it is an early marker of phenotypic changes in response to flow-induced shear (Sakamoto et al., 2010), thermal shock (Sugimoto et al., 2012) or addition of small molecules to the culture medium (Dong et al., 1998; Jeong et al., 2005; Stroka et al., 2012). Changes in phenotype that are not associated with morphological attributes, such as those observed during early neuronal differentiation (Veraitch et al., 2008), can be visualized using fluorescent reporter molecules. However, the gold standard for cell culture characterization remains cell density as it enables the calculation of key proliferation and metabolic rates (Abaci et al., 2010; Cochran et al., 2006), though its determination is often limited to end-point destructive assays.

Quantification of these visual attributes requires either time-consuming and error-prone analysis of digital microscopy images by a human operator, or the use of automated image processing approaches. Software packages such as Cell Profiler (Carpenter et al., 2006) and ImageJ (Schneider et al., 2012) facilitate the establishment of automated image analysis workflows, which typically include a segmentation step that consists in classifying each pixel of an image as either cell or background, enabling the measurement of cellular object features such as size or shape (informing on confluency and morphology, respectively). Segmentation can be facilitated by the use of whole-cell (Machacek and Danuser, 2006; Yu et al., 2010) or nuclei fluorescent markers (Thurnherr et al., 2010). However, the segmentation of images acquired using phase contrast microscopy (PCM), a light microscopy method widely used for the observation of adherent cells in laboratories, poses challenges due to low contrast between cell cytoplasm and cell-free background, and the presence of bright halo artifacts around cellular objects (Otaki, 2000). The misclassification of halo artifacts as cells could artificially inflate cell area measurements and would obfuscate actual cell contours, preventing shape analysis. Segmentation of PCM images thus require specialized algorithms designed to tackle these issues in order to maximize the quality of subsequent measurement of cell characteristics.

To address the low contrast between cytoplasm and background, methods based on the detection of local pixel intensity homogeneity were developed that distinguish cell regions (low homogeneity) from background (high homogeneity) (Theriault et al., 2011; Topman et al., 2011). These approaches are computationally efficient and have high recall (cell pixels tend to be correctly labeled) but also classify halo artifacts as cells, thus lowering the precision of the segmentation. Not discriminating between cellular objects and halo artifacts could result in the overestimation of confluency and the loss of intricate cellular object morphological attributes. This can be remedied by a *post hoc* refinement of the segmentation to correct for halo artifacts using a pattern matching approach. Although segmentation performance was not reported, this approach led to significant improvements for the classification of cell types on PCM images (Bradhurst et al., 2008). Likewise, an approach based on multiple level-set iterations achieved highly accurate detection of cell contours but at the expense of throughput, with a processing time >8 min per image (Ambühl et al., 2012). Alternatively, a method was devised to correct these artifacts prior to segmentation based on models of PCM image formation mechanisms (Yin et al., 2010). However a subsequent study applying this method reported low segmentation performance (Ker et al., 2011). These different approaches have significantly advanced PCM image segmentation but there is still no single method that addresses these challenges in a convenient, reliable and expedient way that is suitable for routine use in the laboratory. Moreover, sampling error due to acquisition of a small number of images or generalization to other cell types, microscope models or imaging conditions continue to be critical issues that require thorough investigation.

Based on the advances in PCM image segmentation made by other groups, we hypothesized that an algorithm based on local contrast thresholding (for a first coarse detection of cellular regions) followed by a rigorous *post hoc* halo correction would enable highly accurate and rapid segmentation of PCM images. Using MATLAB and C++, we then implemented such an algorithm and evaluated its performance. We assessed the algorithm with PCM images of mouse and human embryonic stem cells (mESC and hESC), chinese hamster ovary cells, human neuroblastoma (NB) cells and yeast cells. We also evaluated the impact of varying image acquisition conditions and setups including: microscope manufacturer, camera type (color or black and white), illumination intensity, illumination homogeneity and focusing accuracy. Using the segmentation algorithm, we then analyzed the precision with which the confluency of an entire cell culture can be determined. For this, the precision of confluency determination was compared to that of human estimation and the impact of sampling error on culture confluency measurements was investigated. Confluency determination was then applied to the monitoring of cell responses in various relevant scenarios, such as proliferation, growth arrest, cell death and transient morphological changes. To estimate cell density directly from segmented PCM images, we corrected for the “packing density” of cell colonies by employing basic image features (BIF) for texture analysis (Crosier and Griffin, 2010), and we compared our cell density estimates with results from end-point cell enumeration. Finally, a morphometric analysis and the combination of PCM image segmentation with fluorescence images were applied to the monitoring of early and long-term differentiation events, respectively.

A software toolbox (PHANTAST) containing all algorithms described in the manuscript is made freely available under a permissive open-source license for MATLAB and ImageJ and can be downloaded from http://code.google.com/p/phantast.

## Materials and Methods

### Routine Maintenance of Cells

Mouse ES cells (E14Tg2a, Oct4-GiP, passage number <70, kindly donated by Stem Cell Sciences, Cambridge, UK) were maintained as previously reported (Veraitch et al., 2008).

Chinese hamster ovary cells (CHO-K1, ATCC CCL-61) and human NB cells (SK-N-SH, ATCC CRL-2266) were cultured in T-25 flasks (Fischer Scientific, Loughborough, UK) in Eagle's essential medium (Invitrogen, Paisley, UK) supplemented with 5% fetal bovine serum (Invitrogen).

### Cell Culture Experiments for Confluency Monitoring

Undifferentiated mESC were dissociated and inoculated onto 0.1% (w/v) gelatin-coated tissue culture 6-well plates (Fischer Scientific) at a density of 5 × 10^4^ cells cm^−2^ in 2 mL of medium. Images of ES cell cultures were taken using a motorized, inverted microscope (Nikon Ti-E, Nikon UK Ltd., Kingston Upon Thames, UK). Unless specified differently, 20 random PCM images were acquired per well, at 10× magnification, with a resolution of 1,280 × 960 pixels (Fi-1 color CCD camera, Nikon UK Ltd.). Each image corresponded to a field of view of 1.27 mm × 0.95 mm or ≈1.20 mm^2^.

For the chemical stress experiments, tunicamycin (TM) (Invitrogen) dissolved in DMSO (Invitrogen) was added to the culture medium for a final concentration of 1 µg mL^−1^. Ten random locations per culture were chosen at the beginning of each experiment. Subsequent images during the course of the experiment were taken at the same locations using a motorized microscope stage (Nikon Ltd.).

Environmental stress was induced by removing the 6-well plates from the incubator, and leaving them for 3 h at room temperature (measured to be 20 ± 1°C for all experiments), in a non-controlled gaseous atmosphere and protected from light.

For all other experiments, image acquisition was completed within 10 min (total time of the 6-well plates being outside the incubator).

### End-Point Cell Enumeration

Cell density was determined after each cell culture experiment using an automated cell counter (Vi-Cell, Beckman Coulter, High Wycombe, UK).

### Phase Contrast Image Segmentation

Image processing algorithms were implemented using the MATLAB Image Processing Toolbox (MathWorks, Cambridge, UK) and C++. The algorithms were essentially grouped in two categories: (1) an algorithm to detect the image regions that contain the cells, followed by (2) an algorithm to correct the halo artifacts typical of PCM images. An illustration of the various steps involved is shown in Figure S1.

A MATLAB/C++ implementation of the algorithm as well as GUI tools are freely available under a permissive BSD license (http://code.google.com/p/phantast). A Fiji/ImageJ plugin is also available at the same address.

If necessary, color PCM images were first converted to grayscale images (*I*) by computing a weighted average of the three image channels (weighted 0.30, 0.59, and 0.11 for red, green and blue components respectively, default values for the im2bw function in MATLAB). Local contrast was computed to detect regions of high pixel intensity variations (i.e., regions of the image likely to contain cells). Local contrast (C) was defined as the standard deviation of the image (*I*) within a window (*w*), divided by the mean within the same window. For the window we used a soft-edge Gaussian kernel of standard deviation *σ*. Computation was implemented using the convolution operator (*) according the formula:


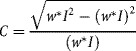


A global local-contrast threshold *ε* was applied to *C* to create a binary image *G* as follows:


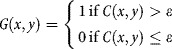


Pixels with a value of 1 represented the cell containing regions. Small holes in *G* (area < *F*_max_) were filled and small objects (area < *R*_max_) were removed.

PCM images exhibit a bright halo (usually between 10 and 30 pixels wide) around cells (Otaki, 2000). Thresholded local contrast tended to classify the entire halo as cell pixels and therefore included the unwanted outer “flank” of the intensity profile. We detected and removed this “flank” using an iterative algorithm that, starting from the borders determined by local contrast thresholding, tracked towards brighter intensities until it reached the interface between the bright halo and the cell edge. This interface was characterized by an abrupt change in gradient direction (Bradhurst et al., 2008).

As a pre-processing step, the direction of the gradient at each location of the image (*I*) was determined using eight Kirsch filters (Fig. S2A). These were tuned to the four cardinal and four inter-cardinal directions. The convolution of each of the Kirsch operators with the image (*I*) was computed. The kernel operation yielding the maximum response for a given pixel determined the gradient direction. These directions were stored for use in the iterative tracking stage of the algorithm. A list of halo locations was initialized with the locations of boundary pixels. In the iterative stage, each halo location was considered until the list was empty. The three pixels arrived at by moving from the current location in the direction of the gradient, and in the two adjacent directions, were considered as candidate halo locations (Fig. S2B). If at least one of these three was a cell pixel, the current location was changed from cell to background. In this case, all candidate halo locations classified as cell pixels were added to the list of halo locations, before removing the current location. If however none of these three pixels were a cell pixel, the current location was confirmed as a cell pixel. In this case, the current location was removed from the list of halo locations and no further pixels were added. During the iterative stage the reduction in object area was tracked and no object was allowed to reduce its area by more than a fraction *A*_ratio_ defined by the user.

### Morphometric Analysis

Morphology of cellular objects was determined based on the binary image resulting from the segmentation process described above. First, objects that were in contact with the border of the image are discarded. A connected-component analysis was then used to identify individual objects. For each object was computed the total area, the solidity (the ratio of the area of the object to the area of its convex hull) as well as the form factor (or shape factor) as follows (Soltys et al., 2005):


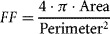


### Cell Density Estimation Using Packing-Corrected Confluency (PCC)

The basic image features (BIFs) of PCM images were computed from the responses of derivative of Gaussian filters as previously described (Crosier and Griffin, 2010; Reichen et al., 2012) using a filter scale (*σ*) of 4 and a flatness threshold (*ε*) of 0. The centroids of the objects corresponding to bright blob features were determined. The centroids that were located outside cell regions (as determined using the segmentation algorithm described above) were discarded. The mean distance between the remaining centroids was computed as the mean value of the Euclidean transform of the binary centroid image. Packing-corrected confluency (PCC) was computed by dividing the image confluency by this distance.

### Augmented Fluorescence Images for GFP Reporter Expression Monitoring

Oct4-GiP mES cells were seeded in 6-well plates at a density of 10,000 cells cm^−2^ and cultured for over 14 days in three distinct culture media: expansion medium (as described in method for cell maintenance above), a spontaneous differentiation medium (expansion medium with 10% FBS and without Leukemia inhibitory factor, LIF), and directed differentiation medium (RHB-A, StemCells Inc., Cambridge, UK). During imaging, nine fields of view across three independent wells were considered per condition. For each field of view, a PCM image and a fluorescence image (FITC/GFP) were acquired using a Nikon Ti-E microscope (Nikon, UK). A CoolLED pE-2 (CoolLED, Andover, UK) was used as excitation source for the fluorescence, enabling the comparison of intensity levels between images (Sato and Murthy, 2012). After segmentation of the PCM images using the method described in “Phase contrast image segmentation,” the cell pixel intensities on the corresponding fluorescence image were used as a basis for the generation of a new image, termed augmented fluorescence image (AFI). Pixels were color-coded as background, cells with no detectable GFP expression, cells with low GFP expression and cells with high GFP expression. The threshold for GFP-positive pixels was set to 0.094 (or 24 for uint8 images), as determined empirically by examining background intensities. Similarly, the threshold to distinguish between low and high expressing cell regions was set to 0.24 (or 60 for uint8 images).

### Generation of Ground Truth Data

Fifty representative images (250 × 250 pixels) were manually processed by a human expert using the Paint.NET software (dotPDN LLC, Kirkland, USA). Each pixel was classified as either cell or background.

### Definition of Segmentation Performance Metrics

Comparison between the algorithm output and the human expert results was done using receiver operator characteristics (ROC) metrics where *TP*/*TN* is the number of true positives/negatives and *FP/FN* is the number of false positives/negatives (with positive referring to cell pixels and negative to background):

Accuracy = (*TP* + *TN*)/((*TP* + *FN*) + (*FP* + *TN*)): the fraction of pixels correctly labeled.
Precision = *TP*/(*TP* + *FP*): the fraction of pixels labelled as cells which are cell pixels.
Recall = *TP*/(*TP* + *FN*): the fraction of cell pixels correctly labeled.
Fscore = 2 × *TP*/(*FP* + *TP*)/((*TP* + *FN*): a measure of the agreement between the algorithm output and the human expert that takes into account both the precision and recall. Values of 0 and 1 signify no and complete overlap, respectively.

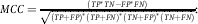
: Matthews correlation coefficient is another metric for the assessment of binary classification problems that is more suitable in case of unbalanced classes. A value of −1 indicates total disagreement between the algorithm and the human expert, 0 that the algorithm is no better than random pixel labelling and 1 represents total agreement between the algorithm and the expert (Powers, 2011).

### Evaluation of Image Segmentation Performance and Parameter Tuning

Parameter optimization was performed by varying each parameter individually; essentially screening an extensive range of parameter sets (over 2 million combinations were explored). The segmentation error was computed as *Δ*_*s*_ = 1 − *MCC*. Leave-one-out cross validation (LOOCV) was employed to assess generalization to unseen images. (See Table S1).

### Confluency Estimation

Image confluency was defined as the fraction of pixels labeled as cell. Culture confluency was estimated by averaging the image confluency computed for at least 20 images taken at random locations.

Let 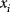 be the confluency as estimated by the algorithm and *x*_*i*_ the confluency determined from the ground truth human annotations, where *i* varies from 1 to *n*, the number of images analyzed. The root mean square error (RMSE) of the algorithm is 
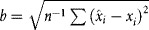
. The mean signed difference (bias) is 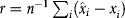. The bias informed on the systematic difference between the mean of repeated measures and the true value. The precision of the algorithmic estimation of confluency is then given by 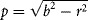. The precision informs on the expected variability associated with the estimation of confluency.

### Confluency Survey

A total of *m* = 7 sets of six images were given to *n* = 14 experienced researchers (>1 year of cell culture experience and used to routine confluency assessment). The *j*th person's estimate of the *i*th image set is *e*_*i,j*_. The mean estimate for the *i*th image is 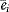. The combined (intra plus inter) variability of the estimated confluency is given by 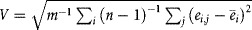. The intra-variability was assessed using the two sets comprising of identical image but with images re-arranged, flipped and rotated. Given *n* pairs of repeated estimates *r*_*α*,1_ and *r*_*α*,2_ by the same expert on the same set of image, the intra-variability is defined as 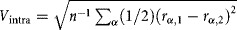. Inter-variability is then estimated as 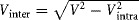.

### Statistical Analysis

Statistical analysis was carried out using MATLAB. All cell culture experiments were performed in triplicate. Unless otherwise specified, results are presented as mean ± SD and Student's independent (unpaired) *t*-test was used to compare means. Means were considered significantly different if the resulting *P*-value was inferior to 0.05. All data was assumed to be normally distributed.

## Results

### Phase Contrast Image Segmentation

**Figure 1:**
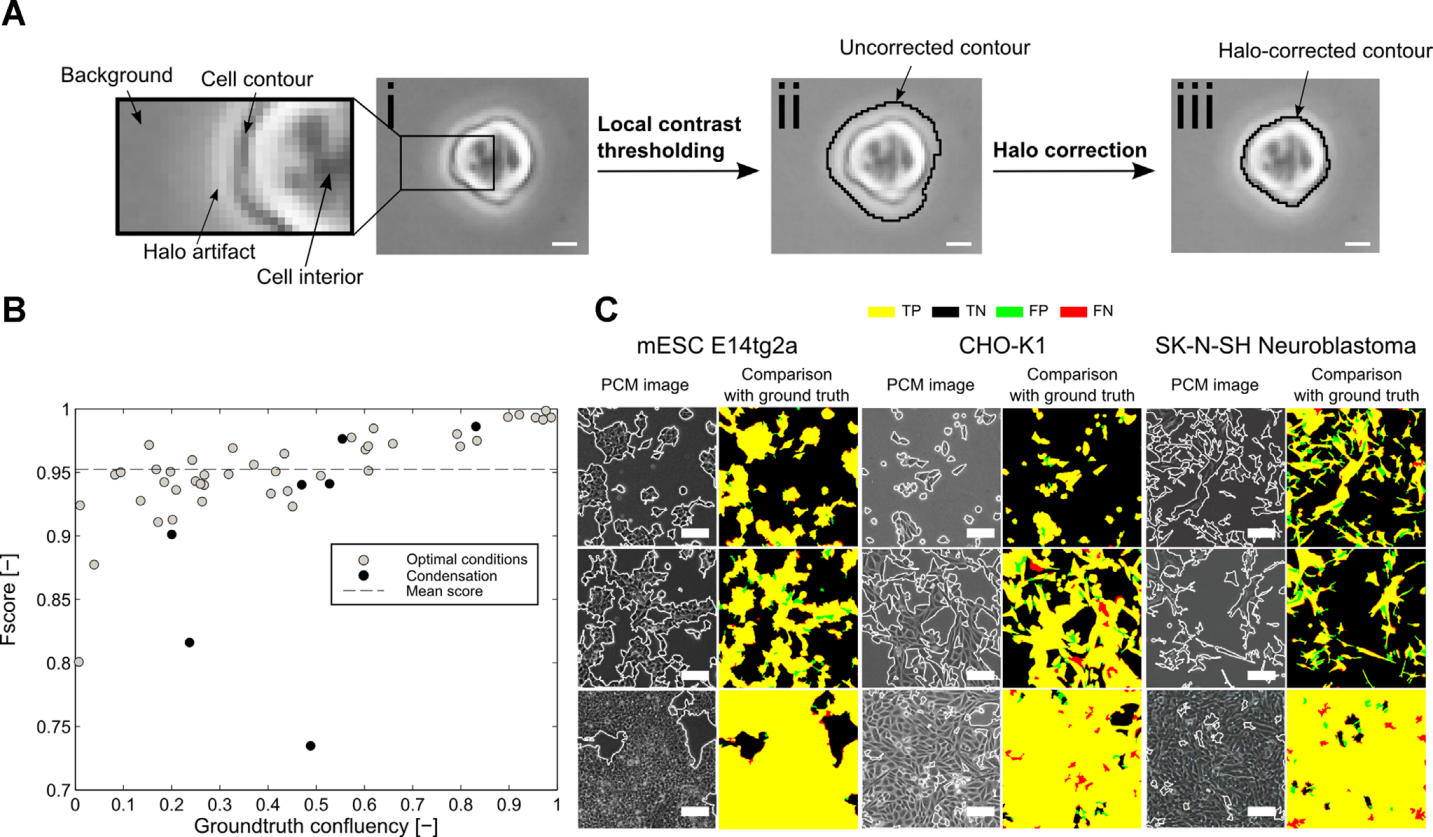
Method for the segmentation of phase contrast microscopy (PCM) images. (A) (i) Cropped region of a mouse embryonic stem cell PCM image shortly after seeding. Insert shows a zoomed-in region with description of the key features of a typical PCM image including a halo artifact surrounding the cell and the lack of contrast between the background and the interior of the cell (ii) Cell contour detected (black line) after local contrast thresholding. A large portion of the pixels corresponding to the halo artifact are incorrectly classified as cell pixels. (iii) Cell contour detected (black line) after post-segmentation halo correction. It conforms to the actual contour of the cell. Scale bars are 10 µm. (B) *F*-score as a function of the ground truth image confluency. Closed symbols represent images with degraded quality due to condensation. (C) Examples of segmentation outcomes for mouse embryonic stem cells (mESC), Chinese hamster ovary cells (CHO) and neuroblastoma cells. For each example, the raw PCM image overlaid with the detected border is compared with the ground truth image (to the right of the raw image). True positives are yellow, false positives green, true negatives black, and false negatives red. Scale bars are 50 µm.

Our method for the automated determination of cell culture characteristics employed a novel image-processing algorithm to accurately and consistently identify cells in phase contrast microscopy (PCM) images ( Fig. 1A). The similarity in pixel intensities between the background and the interior of the cells on PCM images ( Fig. 1A.i) was overcome by the use of local contrast thresholding to identify neighborhoods of pixels presenting large variations in intensity. These regions corresponded to both cells and the halo artifacts ( Fig. 1A.ii). A *post hoc* correction step used the direction of the intensity gradient to exclude halo artifacts and accurately detect cell contours ( Fig. 1A.iii). A detailed description of the segmentation approach is presented in Figure S1. Using a conventional desktop computer (Intel Core I5, 8GB of RAM), our method (PHANTAST) processed images (1,280 × 960 pixels) in less than a second.

A systematic evaluation of the segmentation performance was carried out using cross-validation with 50 mouse embryonic stem cell (mESC) PCM images representative of all stages of a culture (from seeding to full confluence). Average values for all performance metrics considered were high when comparing the outputs of our algorithm with manually annotated ground truths images and were generally higher and less variable than those obtained for a recently described PCM segmentation algorithm (Topman et al., 2011) (Table I). These results were further confirmed by comparison of the segmentation outcomes with live cell membrane fluorescence marker images, which showed a good agreement (Fig. S3). A decrease in segmentation performance was measured for low confluency images that corresponded to the early stage of a culture, due to small intricate structures ( Fig. 1B).

**Table I:** Evaluation of segmentation performance

	Metric	Mean	SD	95% CI
Our method	F-score	0.94	0.05	[0.93, 0.96]
	Precision	0.96	0.04	[0.94, 0.97]
	Recall/sensitivity	0.94	0.07	[0.92, 0.96]
	Accuracy	0.97	0.03	[0.96, 0.98]
	MCC	0.88	0.14	[0.84, 0.92]
Topman et al. (2011)	F-score	0.84	0.11	[0.61, 1.06]
	Precision	0.75	0.16	[0.44, 1.06]
	Recall/sensitivity	0.97	0.03	[0.91, 1.04]
	Accuracy	0.90	0.04	[0.81, 0.99]
	MCC	0.70	0.13	[0.44, 0.97]

Results obtained using a leave-one out cross validation (LOOCV) on 50 images for the *F*-score, precision, recall, accuracy and Matthews correlation coefficient (MCC). For comparison purposes, the same approach was applied to a previously described PCM image segmentation algorithm (Topman et al., 2011). The results are shown as the mean across the 50 images, the standard deviation and the 95% confidence interval.

Using the same parameters than those determined during cross-validation with mESC images, we assessed the segmentation performance for two other mammalian cell lines, namely Chinese hamster ovary (CHO) cells, which are widely used in production of therapeutic recombinant proteins (Chu and Robinson, 2001), and human neuroblastoma (NB) cells, frequently employed as a model for in vitro study of neurotoxicity and neurodegeneration (Cheung et al., 2009). PHANTAST successfully detected structures that are not commonly attributed to mESC, such as dendritic projections and flat cell bodies, resulting in mean *F*-scores of 0.95 ± 0.03 and 0.90 ± 0.07 for CHO and NB cells, respectively ( Fig. 1C). These high segmentation scores indicated that the optimal parameters determined for mESC constitute a reasonable starting point for other cell types. Since the determination of optimal segmentation parameters for a particular cell line or imaging setup can be tedious and time consuming, we devised simple protocols and a graphical user interface to facilitate this process (Fig. S5). In addition, encouraging preliminary results with a wide variety of other cell types, including human embryonic stem cells, NIH/3T3 and yeasts showcased the broad applicability of the proposed segmentation algorithm (Fig. S4).

**Figure 2:**
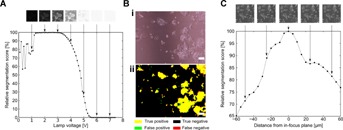
Tolerance of the segmentation algorithm to variations in imaging conditions. (A) Effect of the illumination intensity on the segmentation quality (as assessed using the *F*-score). Lamp intensities from 0 to 7 V were tested. (B) Example of PHANTAST segmentation outcome for a PCM image with inhomogeneous illumination patterns as caused by the presence of a liquid meniscus in the light path. Scale bar is 100 µm. (C) Effect of the distance from the in-focus plane on the segmentation quality (as assessed using the *F*-score). The in-focus was determined visually by an experienced microscope user. The focus was changed in steps of 5 µm using a Piezo *Z*-stage.

**Figure 3:**
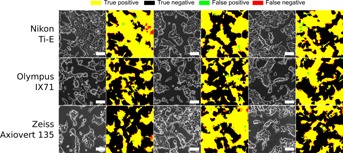
Comparison of segmentation performance for images of a single Oct4-GiP mESC culture acquired using different phase contrast microscopes, cameras and imaging protocols. The microscopes used were a Nikon Ti-E microscope (Fi-1 color camera), a Olympus IX71 (Hamamatsu ORCA-ER C4742-80-12AG monochrome camera) and a Zeiss Axiovert 135 (Hamamatsu ORCA-R2 C10600-10B-H monochrome camera). Each row is a different microscope. Three fields of view per microscope were considered. The raw phase contrast microscopy image is shown with the segmentation result overlaid in white. Next to it is the comparison with the manually annotated ground truth image. All processing parameters were kept constant. Scale bars are 100 µm.

The tolerance of the algorithm to variations in imaging conditions was assessed. Variations in illumination intensity did not impact segmentation performance for intensities within the range of values typically used for routine observation ( Fig. 2A). Similarly, illumination patterns, such as those caused by liquid menisci in small-scale devices, did not noticeably affect the quality of the segmentation ( Fig. 2B). In contrast, any deviation from ideal focusing was found to have an impact on the segmentation performance ( Fig. 2C). The formation of a condensation layer is another issue frequently encountered during imaging of live cells due to the temperature difference between the incubator and the imaging environment. Condensation impacts image quality by decreasing the overall contrast of an image. This had varying effects on the segmentation outcome, depending on the severity of the decrease in image quality ( Fig. 1B). We also investigated how variations in the imaging setup affected the segmentation quality by imaging the same culture of Oct4-GiP mESC with three different microscopes and cameras, including color and b/w cameras ( Fig. 3). A one-way ANOVA showed that the choice of the imaging setup did not have a statistically significant effect on the segmentation performance as assessed using the F-score (df = 2, *F* = 2.75, *P*-value = 0.14).

### Estimation of Culture Confluency

Confluency determination through visual inspection is subjective and prone to high inter-individual variability (Topman et al., 2011). This was further confirmed by a survey we conducted showing that the inter- and intra-researcher variability from 14 experienced cell culture researchers amounted to 9.5% and 6.8%, respectively, yielding a combined variability (i.e., the precision of the researchers' estimations) of 11.7% (Fig. S6).

When using PHANTAST, the confluency of a single image was determined directly from the segmentation outcome by computing the fraction of an image that was labelled as cell pixels. The precision for image confluency determination was found to be 2.7% and the quality of that estimation was consistent for the entire range of confluencies (Fig. S7A). When the *post hoc* halo correction step was omitted, the precision of the estimation more than doubled to 7.1%, which highlighted the importance of this step (Fig. S7B).

When estimating the confluency of a whole culture, it is necessary to account for the uncertainty introduced by random sampling of a non-uniformly distributed cell population (Dehlinger et al., 2013; Usaj et al., 2011), as it is not practical to acquire the large number of images necessary to cover the entire culture area of vessels commonly used for adherent cell culture (Table S2). For the area of a well of a 6-well plate (∼9.6 cm^2^), we acquired 20 images (at 10× magnification) at random locations, resulting in a sampling error of 1.74% (Fig. S8). When combined with the precision of the confluency estimation based on a single image (2.7%), we obtained an overall precision of 3.2%. Although obtained from a larger set of images, this precision represents a ∼3.5-fold improvement over the variability estimated from our survey. Additionally, with the measurement time being comparable to that of visual inspection (the algorithm took less than a minute to process the 20 images), this further highlights the value of automated image processing routines, and underscores the high performance of our segmentation algorithm.

### Mouse Embryonic Stem Cell Culture Monitoring

**Figure 4:**
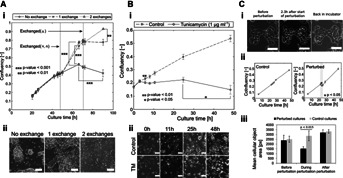
Monitoring of mESC cultures. (A) (i) Time course study of the effect of medium exchange on confluency. Twenty random PCM images per well (of a 6-well plate), at 10× magnification, were used for confluency determination using PHANTAST. Confluency is determined with high precision in <5 min. Data shown as mean ± SD (across 3 wells). (ii) Representative PCM images of the cultures after 90 h. Scale bars are 100 µm. (B) (i) Time course study of the effect of a chemical stressor (TM, 1 µg mL^−1^) on mESC growth. 10 locations were used per well (of a 6-well plate), at 10× magnification, for confluency determination using PHANTAST. These locations were imaged for 50 h. Data shown as mean ± SD (across 3 wells). (ii) PCM images of treated cultures and controls at different time points. Scale bars are 100 µm. (C) (i) PCM images illustrating morphological changes in response to environmental stress. Annotations indicate the time since the beginning of the perturbation. Scale bars are 100 µm. (ii) Monitoring of mESC response to environmental stress. 20 random PCM images per well (of a 6-well plate), at 10× magnification, were used for confluency determination using PHANTAST. Data shown as mean ± SD (across 3 wells). (iii) Effect of the perturbation on the mean cellular object area (includes both single cells and colonies). Data shown as mean ± SD (across 3 wells).

The application of confluency determination to non-invasive monitoring of adherent cell cultures was demonstrated by investigating various experimental scenarios. These scenarios were chosen to include a variety of measureable cell responses, including rate of proliferation, growth arrest, cell death and morphological changes ( Fig. 4).

Confluency of mESC cultures under three different medium exchange schedules was monitored: no exchanges, one exchange (48 h), and two exchanges (48 and 70 h; Fig. 4A.i). The resulting confluency profiles confirmed the intuition that medium exchanges would promote cell proliferation. Indeed, end point analysis showed that the number of medium exchanges had a significant effect on confluency (One-way ANOVA, *P*-value = 3.01 × 10^−6^). This result was further confirmed by end-point cell density determination (One-way ANOVA, *P*-value = 3.21 × 10^−5^). The gradual decline in confluency measured for the cultures with no medium exchange starting at ∼60 h into the cultures suggested a loss of cell viability potentially due to nutrient deprivation or toxic metabolites accumulation. This was in agreement with the presence of apoptotic bodies in the culture medium ( Fig. 4A.ii). These results indicated that continual confluency monitoring could be used to non-destructively assess cell growth and also serve as an early warning system based on the detection of unexpected growth patterns.

Confluency monitoring was also employed to determine cell response to the addition of tunicamycin (TM) to the culture medium. TM is a mixture of antibiotics commonly employed to study the unfolded protein response (Tabas and Ron, 2011), and is also thought to play a role in ES cells self-renewal and differentiation (Blanco-Gelaz et al., 2010; Cho et al., 2009). Cells were imaged over a period of 48 h after addition of TM to the culture medium ( Fig. 4B.i). The confluency of the treated cultures hit a plateau after 6 h and remained constant until a decrease was measured after 48 h, which was accompanied by a large number of apoptotic bodies in suspension ( Fig. 4B.ii). This response of cells to TM, growth arrest followed by cell death, was consistent with the current understanding of the mechanisms underlying endoplasmic reticulum stress (Yoshida, 2007), demonstrating the ability of confluency monitoring to unravel multi-stage cell response to toxic compounds.

Confluency also informs on changes in morphology when those are accompanied with variations in cell area. Thermal shock was investigated by leaving the cells at room temperature and without CO_2_ control, inducing morphological changes that could be reversed by restoring normal growth conditions (37°C, 5% CO_2_), as shown by time-lapse imaging ( Fig. 4C.i). Culture confluency monitoring showed that a significant effect was detected for the duration of the perturbation ( Fig. 4C.ii). Moreover, the perturbation induced a statistically significant decrease in cellular objects area when compared to control cultures. No difference was detectable prior to the perturbation and after restoring normal growth conditions ( Fig. 4C.iii).

### Cell Density Estimation

**Figure 5:**
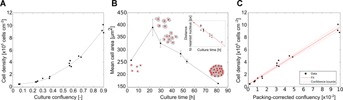
Cell density estimation of mESC cultures based on PCM images. (A) Relationship (adjusted *r*^2^ = 0.89) between cell density (as measured after detachment) and culture confluency (determined using PHANTAST). (B) Changes in mean cell area during a typical mESC culture. Cells were counted using a live nuclear stain (Hoechst 33342). The insert shows the change in distance between nuclei during the same culture. (C) The relationship between cell density (as determined after detachment) and packing-corrected confluency (PCC), computed from the confluency and the distance to nearest nucleus-like feature using PHANTAST.

For mESC, and colony-forming cell lines in general, cell density as determined using sacrificial counting methods is not linearly proportional to culture confluency ( Fig. 5A). Indeed, high variations in the area of individual cells were measured during the course of a culture ( Fig. 5B), consistent with typical pluripotent stem cells growth behavior (Harb et al., 2008). In general, confluency is therefore a poor predictor of cell density.

We hypothesized that confluency could be corrected in order to account for these changes in cell area by using the mean distance between cell nuclei, which was found to linearly decrease as the culture progressed ( Fig. 5B insert). However, this distance can only be directly determined by using nuclei fluorescent markers. Alternatively, texture analysis of unlabeled PCM images enabled the use of blob-like features as surrogates for the estimation of the distance between cells. The packing-corrected confluency (PCC) was then computed by dividing the image confluency by the distance between blob-like objects. PCC was found to increase linearly with cell density, as shown by an adjusted *r*^2^ of 0.983 ( Fig. 5C).

In order to determine cell density of a culture using this method, it is necessary to first perform a calibration step by relating PCC with cell density measurements in order to determine regression coefficients. Generalization of this approach was assessed with three mESC cultures in 6-well plates spanning the whole range of possible confluencies, where one culture was used for calibration and the remaining two for performance evaluation. This process was repeated three times, so that all cultures were used for calibration once. The normalized root mean square error (NRMSE) of the cell density estimation decreased from 21.5% when using confluency to 10.2% for PCC (Table S3). PCC was thus found to be a good predictor of mESC cell density.

### Monitoring Phenotypic Changes During Differentiation

**Figure 6:**
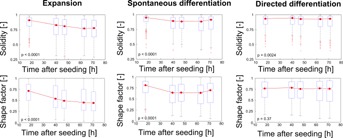
Morphometric analysis of early differentiation events. Cells were cultured in three medium formulations promoting pluripotency maintenance and expansion, spontaneous differentiation or directed differentiation. The solidity (a measure of the convexity) and shape factor of cellular objects were computed after initial segmentation using PHANTAST. Objects in contact with the border of the image were omitted. Each data point shows the distribution of object statistics computed using 9 images, across 3 wells of a 6-well plate. For each box, the central red mark is the median, the edges are the 25th and 75th percentiles and the whiskers extend to the most extreme data points (not including outliers). The “+” markers represent outliers, values outside of the range [75th quartile − 1.5 × (75th quartile − 25th quartile); 75th quartile + 1.5 × (75th quartile − 25th quartile)].The *P*-values are computed using one-way ANOVA with the morphological attribute as the dependant variable and time as the independent variable.

Automated image processing using PHANTAST was used to monitor the phenotypic changes that occurred when mESC were cultured in three different culture medium formulations: expansion medium that supports the maintenance of pluripotency, spontaneous differentiation medium, and medium for directed differentiation toward neuronal lineages. First, early morphological changes (<70 h after seeding) were monitored using solidity (a measure of convexity) and the shape (or form) factor, a metric used to characterize the shape of objects (Belletti et al., 2008) ( Fig. 6). During expansion, the median solidity and shape factor decreased overtime and plateaued after 60 h. Similar profiles were measured for cells in spontaneous differentiation medium, albeit with slightly higher initial levels and a stabilization already occurring after only 40 h. For both these conditions, time was found to have a significant effect on the two morphological attributes. In contrast, the solidity and shape factor of cells in directed differentiation medium were approximately constant for the whole duration of the experiment.

The morphometric analysis was limited to early differentiation events due to the formation of large colonies that would fill a large portion of the field of view, preventing the detection of their contours and thus the determination of morphological attributes at later stages of the process. In order to investigate changes in phenotype during differentiation, PCM image segmentation was used in combination with fluorescence microscopy to monitor the changes in expression patterns of the Oct4 pluripotency marker for 14 days. The reporter mESC line Oct4-GiP expressed GFP under the direction of regulatory elements of the mouse *Oct4* gene, allowing to relate GFP expression levels to cell pluripotency (Ying et al., 2003).

**Figure 7:**
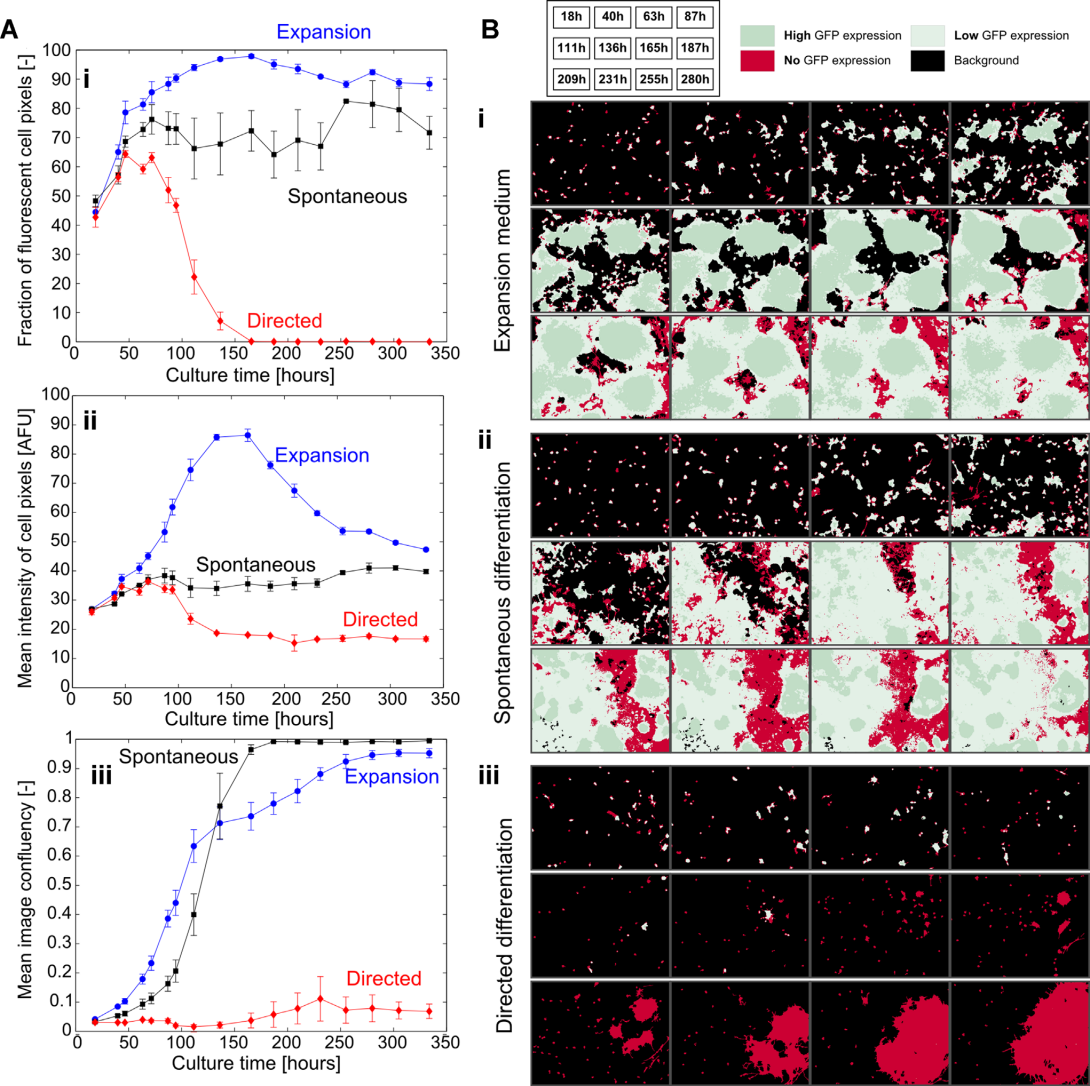
Long-term non-invasive monitoring of GFP expression patterns. (A) Time course measurements of the fraction of fluorescent cell pixels (i), mean fluorescence intensity of cell pixels (ii) and mean image confluency (iii). Oct4-GiP mES cells were cultured in 6-well plates in three different media formulation (expansion in blue, spontaneous differentiation in black and directed differentiation in red). Each data point is the mean of three field of views per well, across three wells. Error bars are the standard deviation. (B) Augmented Fluorescence Images (AFI) of Oct4-GiP mES cells in 6-well plates cultured in different media formulations: (i) expansion, (ii) spontaneous differentiation and (iii) directed differentiation. Green represents fluorescent cell pixels (dark green is high expression, light green is low expression), red indicates non-expressing cell pixels and black is background (non-cell) pixels. The time corresponding to each image is shown in the insert at the top.

Augmented fluorescence images (AFIs) were generated by combining the information related to cell position as given by PCM segmentation with intensity values from a fluorescence image of the same field of view. AFIs are abstracted representation of fluorescence patterns where regions are classified as background, negative cells, low GFP expressing cells and high GFP expressing cells (Fig. S9a.A). In addition to the confluency determined from the PCM segmentation ( Fig. 7A.iii), this approach enabled the quantification of the fraction of GFP-positive cell pixels ( Fig. 7A.i) and that of the mean fluorescence intensity of cell pixels ( Fig. 7A.ii), yielding results that were consistent with end-point FACS analysis (Fig. S9B).

For all three conditions tested, the fraction of fluorescent cell pixels was about 50% 18 h after seeding and increased as the culture progressed. This trend indicated that GFP content of some cells was too low to be detected as positive until sufficient accumulation had occurred. This is consistent with the results of a previous study where a large fraction of cells were classified as low GFP producers shortly after seeding (Veraitch et al., 2008).

Beyond 50 h of culture, the fraction of GFP-positive cell pixels in expansion medium consistently remained between 80% and 100% as the cells formed large, high expressing colonies ( Fig. 7B.i). After 7 days of culture, GFP-negative cells had started colonizing available space between these colonies, accompanied by both a decrease in mean fluorescence intensity ( Fig. 7A.ii) and a surge in image confluency ( Fig. 7A.iii), indicating a loss of pluripotency most likely caused by overgrowth. Indeed, GFP-negative regions corresponded to flat cells, a morphology typical of somatic cells (Fig. S10A). Cells in spontaneous differentiation medium grew mostly in low-expressing colonies with a significant fraction of negative cells observed as early as 5 days after seeding ( Fig. 7B.ii). The fraction of GFP-positive cell pixels stabilized at around 70% and the mean fluorescence intensity of cell pixels also reached a plateau at about a third of the maximum intensity achieved in expansion medium. Again, GFP-negative regions corresponded to cells with a somatic morphology (Fig. S10B). When using directed differentiation medium, the cells did not form colonies and remained either low-expressing or negative until 7 days into the culture where rapid expansion of negative colonies was observed ( Fig. 7B.iii), as indicated by an increase in confluency. The fraction of GFP positive cell pixels started to decline after 4 days into the culture and reached 0% shortly after 6 days. The fluorescence intensity of cell pixels decreased toward levels close to that of the background in the same time frame. Morphology of the cells strongly suggested differentiation towards neuronal lineages, which was consistent with the absence of GFP signal (Fig. S10C).

## Discussion

**Figure 8:**
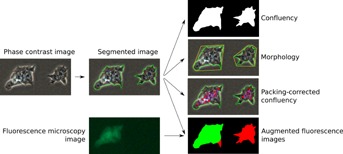
Summary of the proposed method PCM images were first segmented using local contrast thresholding and post hoc halo correction. Confluency could be determined directly from the outcome of the segmentation. Morphological analysis of cellular objects was carried out using convexity and shape factor as metrics. Cell density could be estimated using packing-corrected confluency, a metric based both on confluency and the mean distance between blob-like texture features. Finally, PCM segmentation was combined with fluorescence imaging data to enable the determination of temporal and spatial fluorescence patterns.

We set out to develop an imaging-based method for the determination of adherent cell culture characteristics that is convenient, quick and very precise ( Fig. 8). Based solely on unlabelled PCM images, it can be used to determine culture confluency, estimate cell density and measure morphological attributes. Moreover, it can be used as a tool to facilitate the interpretation of fluorescence microscopy data.

At its heart is the segmentation algorithm that enables the creation of automated image analysis workflows in MATLAB or ImageJ despite the challenges usually associated with PCM image processing. High segmentation performance was reported for three mammalian cell lines with vastly different visual features. Moreover, this level of performance was consistent for all stages of a culture and regardless of the model of microscope or type of camera used, the illumination intensity and the presence of illumination patterns. These results suggest that our algorithm accommodates non-ideal imaging conditions and that it produces results that can be compared across trials and laboratories.

The quality of the segmentation directly impacted the ability to produce reliable culture characteristics measurements, as highlighted by a 2.5-fold improvement in the precision of image confluency determination when using a *post hoc* halo correction. The determination of the confluency of a whole culture as opposed to that of a single image involves sampling multiple locations of the growth area, thus introducing an additional source of variability. Whereas previous studies employed three images or less for a culture area of 9.6 cm^2^ (Ker et al., 2011; Topman et al., 2011), we determined that 20 random images were necessary to strike a reasonable and practical balance between throughput (imaging time) and quality of the measurement. When comparing with our survey, this meant a 3.6-fold increase in precision over human estimation. The number of images to use will depend on many factors, including the field of view of the camera, the cell line used and the homogeneity of the seeding. Nevertheless, the sampling error using 20 images was negligible in comparison to the confluency estimation error per image and thus constitutes a reasonable starting point for other conditions.

The robustness of the culture confluency measurements was further demonstrated by the high reproducibility across trials achieved when monitoring mESC culture during expansion, after the addition of a toxic compound to the culture medium and in response to an environmental shock, three scenarios that are highly relevant to stem cell applications: optimization of expansion protocols for the generation of large quantities of therapeutic-grade cells (Csaszar et al., 2012), drug discovery and cell-based toxicity assays (Scott et al., 2013). In all three cases, the data generated using PHANTAST helped to gain insight into the dynamics of the studied process solely based on unlabeled PCM images.

Significant progress toward the establishment of a method for cell density estimation based on light microscopy images was made by combining confluency measurements with texture analysis. Indeed, we introduced a novel metric termed packing-corrected confluency (PCC), which was shown to be linearly correlated with cell density as determined after detachment, despite large variations in cell area due to the formation of colonies in the case of mESC cultures. PCC effectively minimized the effects of area variability, resulting in NRMSE values comparable to previously reported figure for confluency-based estimation of cell count for cell lines which undergo only small variations in size during culture growth (9% and 10% for C2C12 and 3T3-L1, respectively) (Topman et al., 2011). Unlike confluency determination, however, this method requires calibration data. Further investigation are required to determine how applicable a calibration remains when cell culture conditions change.

We also demonstrated how PCM image segmentation could be leveraged for the monitoring of mESC differentiation through both morphometric analysis and in combination with fluorescence microscopy. The measured profiles for the solidity and shape factor were different depending on the culture medium formulation, indicating that our algorithm might be applicable to studies on the relationship between morphology and cell fate (Matsuoka et al., 2013). The early differentiation stage analysis was supplemented with long-term monitoring by combining PCM segmentation and fluorescence microscopy images of a GFP pluripotency reporter. The PCM segmentation step was used to determine cell locations and thus provided a context for the interpretation and quantification of the fluorescence data. It essentially replaced the use of a whole-cell fluorescent marker (Ng et al., 2010; Pasquier et al., 2012), consequently reducing unnecessary culture handling and freeing a fluorescent channel, allowing for the imaging of additional fluorophores. The generation of augmented fluorescence images (AFIs) enabled the analysis of temporal expression profiles in addition to simplifying the interpretation of spatial patterns by abstracting the fluorescence data.

In summary, we designed an algorithm that segmented PCM images with high performance in a wide range of conditions and for different cell types. Combined with other imaging routines, it produced high quality measurements of key adherent cell culture characteristics with very short processing times. All the algorithms were bundled in a software toolbox (PHANTAST). Though we provide implementations with graphical user interfaces in MATLAB, ImageJ and as a standalone tool, the open-source license used is permissive and allows for integration in other image processing packages as well as commercial solutions. Since empirical parameter tweaking is tedious, and thus a significant barrier to adoption for image processing algorithms (Pretorius et al., 2011), we also developed a tool that facilitates the determination of optimal segmentation parameters. PHANTAST is therefore a very robust and convenient tool to generate quantitative data from cell culture experiments without the need to detach cells. In addition, it can serve as the first critical step in advanced image processing workflows, such as pattern recognition. Its precision and low-processing time also make it suitable for the integration with automated cell handling systems that are currently being developed for the manufacturing of stem cell-derived therapeutic cells (Thomas et al., 2008). Preliminary steps were taken in this direction with the integration of PHANTAST in a LabVIEW routine for the automated, online monitoring of culture confluency in a microfabricated bioreactor (Fig. S11).

## Supporting Information

**Figure S1.** Illustration of the image segmentation process. (**A**) Typical phase contrast microscopy (PCM) image (i) and corresponding intensity profile (ii) along the dashed black line. (**B**) Image after application of local contrast filter with *σ* = 1.4 (i) and the corresponding intensity profile (ii) along the dashed white line. (**C**) Binary mask obtained after thresholding of the local contrast image with *ε* = 0.06; a pixel value of 1 (white) represents cells (cell mask), 0 (black) represents the background (i); (ii) shows the original image overlaid with the boundary from the binary mask of (i). (**D**) Illustration of the iterative halo correction process. A subset of the image of (C, ii) is magnified and the pixels are overlaid with arrows representing the direction of the pixel intensity gradient. The first iteration (D, i) starts at the boundary pixels of the binary mask obtained after thresholding. For ease of understanding, only one starting pixel is considered for each iteration (represented as a green triangle). Valid pixel candidates (i.e., pixels for which the gradient direction points to the white “cell part” of the binary mask) are indicated by green circles while invalid candidates (e.g., pixels outside the white cell mask) are shown as red squares. The binary mask is shrunk (ii shows an intermediate step of the process) until it reaches the contour of the cells (i.e., no more valid candidates) as shown in iii. (E) Final output of the image processing algorithm overlaid on the original PCM image of (A, i).

**Figure S2.** Kirsch kernels for gradient direction computation. (**A**) Kirsch kernels employed for gradient direction determination. (**B**) Gradient directions (black arrows) were associated with their closest directions (grey-filled squares): the pixel in the direction of the gradient, and the two pixels in the two adjacent directions.

**Figure S3.** Comparison of segmentation outcome using PHANTAST with a cell membrane fluorescent marker. The top row contains PCM images at three different magnifications overlaid with the segmentation result (white). The bottom row shows the fluorescence image of the cell membrane marker (CellMask) with the same overlay (green).

**Figure S4.** Segmentation results for a wide range of cell types. For each set, the first image is the raw PCM image and the second is the same raw imaged overlaid with the segmentation result in green. (**A**) NIH/3T3, adapted from Cell: An Image Library (John Elliot, CIL 7883). (**B**) *Saccharomyces cerevisiae*, adapted from Cell: An Image Library (David Ball, Jean Peccoud, CIL 39626). (**C**) Human induced pluripotent stem cells cultured on Matrigel, courtesy of Zuming Tang (University College London, Biochemical Engineering). (**D**) Human embryonic stem cell colony on Matrigel, adapted from Cell: An Image Library (Sabrina Lin, Prue Talbot, CIL 12626). (**E**) Pichia pastoris, courtesy of Dr Rochelle Aw (Imperial College London, Division of Molecular Biosciences). (**F**) Human sperm, adapted from Cell: An Image Library (Michael Crammer, CIL 34524). (**G**) Acanthamoeba castellani, adapted from Cell: An Image Library (Thelma Dunnebacke, CIL 19326).

**Figure S5.** Parameter optimization protocols using (**A**) the live mode of PHANTAST or (**B**) the optimization tool.

**Figure S6.** Confluency estimations by 14 experienced human experts based on seven sets of six phase contrast microscopy (PCM) images acquired during mESC expansion. Each researcher was shown seven sets of PCM images. Each set contained six images acquired at random locations within a single well. Each individual was asked to estimate the confluency for each set based on the six images in the set. Six of the seven sets were taken at different time points to represent stages of the expansion process (from shortly after cell seeding to near confluency). A seventh set (30') contained the same images than the ones from the 30 h time point, but its images had all been rotated by 90°, mirrored, and were displayed in a different arrangement. Thus, effectively, two sets of images existed which had identical confluency (images taken at 30 h into the culture). To avoid a bias when estimating the confluency, the sets were not presented in chronological order. To ensure that each individual was faced with the same task, the seven sets were presented in the same order to all researchers. For each box, the central mark is the median, the edges are the 25th and 75th percentiles and the whiskers extend to the most extreme data points (not including outliers). The “+” markers represent outliers. These results highlight the high variability of confluency estimations made by human operators.

**Figure S7.** Confluency estimation performance for single images assessed using 50 mESC images. (**A**) With halo correction: The mean signed difference (bias) for 50 confluency estimates was −0.35% with a 95% confidence interval (CI) of [−1.12%, 0.42%]. The root mean square error (RMSE) of the estimates was 2.69% and the precision, as determined from the RMSE and the bias, was 2.67%. (**B**) Without halo correction: The mean signed difference (bias) for 50 confluency estimates was 4.78% with a 95% confidence interval (CI) of [2.72%, 6.82%]. The RMSE of the estimates was 8.59% and the precision, as determined from the RMSE and the bias, was 7.14%.

**Figure S8.** Estimation of the error due to finite sample size. One Hundred images of a mESC culture were acquired and processed. The mean confluency and the corresponding 95% confidence interval were computed. The standard deviation obtained using 100 images can be used to compute the confidence interval length for various sample size as follow: 1.96 * *σ*/√*n* where *σ* is the standard deviation for 100 images and n is the sample size. For comparison purposes only, the intrinsic precision of the algorithm is plotted (dashed lined) in the same graph.

**Figure S9.** Determination of spatial and temporal expression pattern of a fluorescent reporter. (**A**) Combination of a segmented phase contrast image (i) with a fluorescence image, here with the segmentation overlaid in white (ii) in order to generate an abstract representation showing expressing regions (light and dark green for low and high expression, respectively), regions with cells that do no express the reporter (red) and background with no cells (black). (**B**) Comparison of the results of the determination of GFP+ cells using the imaging method described in A with flow cytometry.

**Figure S10.** Phase contrast images of mESC cells after 14 days of culture in (**A**) expansion medium with LIF, (**B**) spontaneous differentiation medium without LIF, and (**C**) directed differentiation medium.

**Figure S11.** Mouse embryonic stem cell growth monitoring in a microfabricated bioreactor. (a) Confluency measurements for a single field of view imaged over 5 days. An image was automatically acquired every 30 min. (b) Phase contrast images acquired at the time points indicated in a (first row) and images overlaid with the contours detected by PHANTAST (second row). Scale bars are 100 µm.

**Table SI.** Optimised parameters for the segmentation algorithm. The investigated range refers to the range of values screened during the optimization process.

**Table SII.** Number of images required to cover the whole culture area of various commonly used culture vessels. These calculations are based on a field of view of approximately 1.2 mm^2^.

**Table SIII.** Cross-validation of the cell density estimation method based on three cultures in 6 well-plates that spanned the whole range of possible confluencies. A linear regression between packing-corrected confluency (PCC) and cell density after counting was done for one culture and used to predict the cell density of the other 2. For comparison purposes, the same validation was done by using confluency instead of PCC.
