# Supplementary figures and images for: Automated Method for the Rapid and Precise Estimation of Adherent Cell Culture Characteristics from Phase Contrast Microscopy Images
Source: Biotechnol Bioeng. 2013 Oct 5;111(3):504–17. doi: 10.1002/bit.25115 (PMC4260842; doi:10.1002/bit.25115)

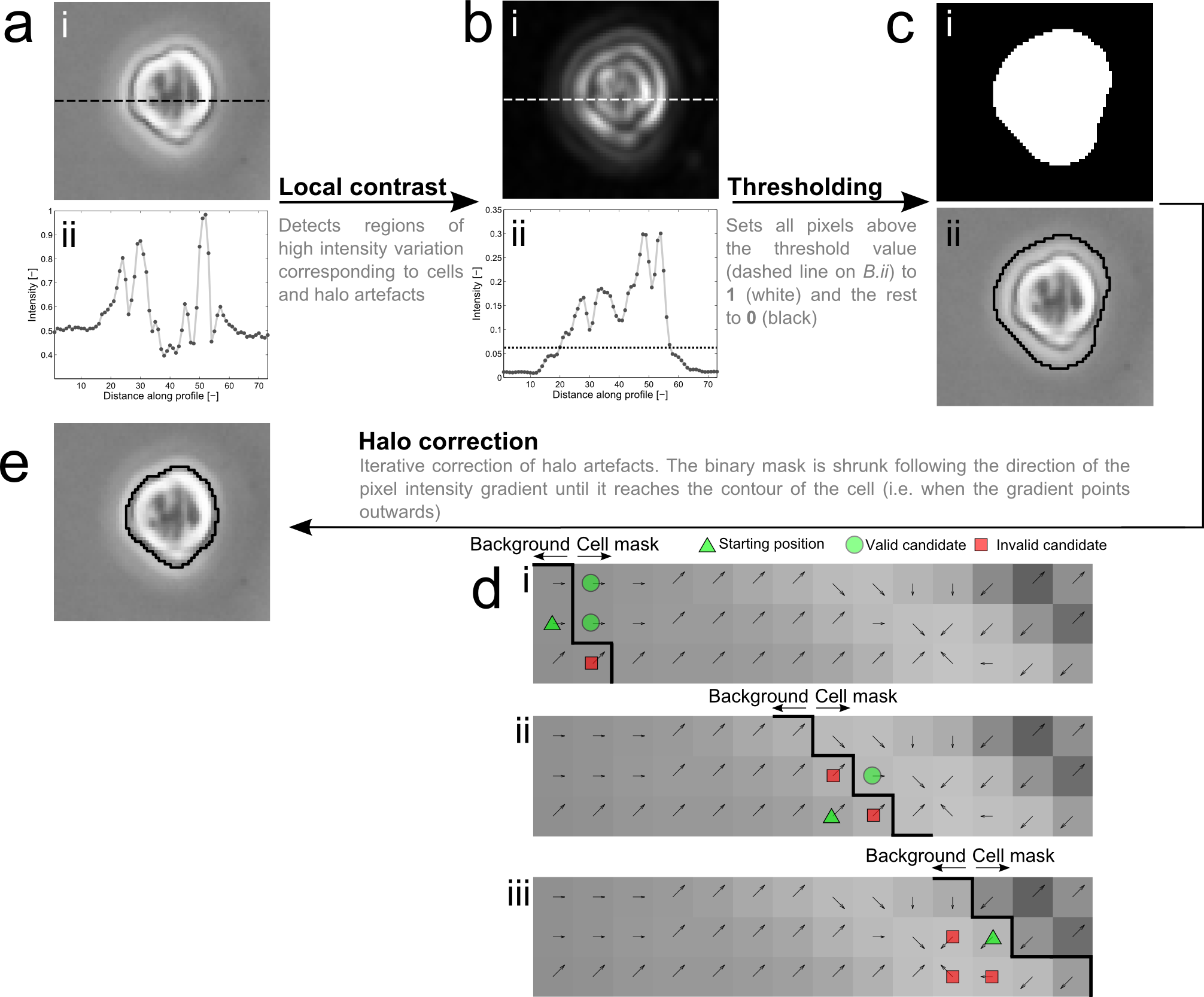

Supplement: Supplementary file 1 — Figure S1. Illustration of the image segmentation process. (A) Typical phase contrast microscopy (PCM) image (i) and corresponding intensity profile (ii) along the dashed black line. (B) Image after application of local contrast filter with σ = 1.4 (i) and the corresponding intensity profile (ii) along the dashed white line. (C) Binary mask obtained after thresholding of the local contrast image with ε = 0.06; a pixel value of 1 (white) represents cells (cell mask), 0 (black) represents the background (i); (ii) shows the original image overlaid with the boundary from the binary mask of (i). (D) Illustration of the iterative halo correction process. A subset of the image of (C, ii) is magnified and the pixels are overlaid with arrows representing the direction of the pixel intensity gradient. The first iteration (D, i) starts at the boundary pixels of the binary mask obtained after thresholding. For ease of understanding, only one starting pixel is considered for each iteration (represented as a green triangle). Valid pixel candidates (i.e., pixels for which the gradient direction points to the white “cell part” of the binary mask) are indicated by green circles while invalid candidates (e.g., pixels outside the white cell mask) are shown as red squares. The binary mask is shrunk (ii shows an intermediate step of the process) until it reaches the contour of the cells (i.e., no more valid candidates) as shown in iii. (E) Final output of the image processing algorithm overlaid on the original PCM image of (A, i). [file bit0111-0504-SD1.tif]

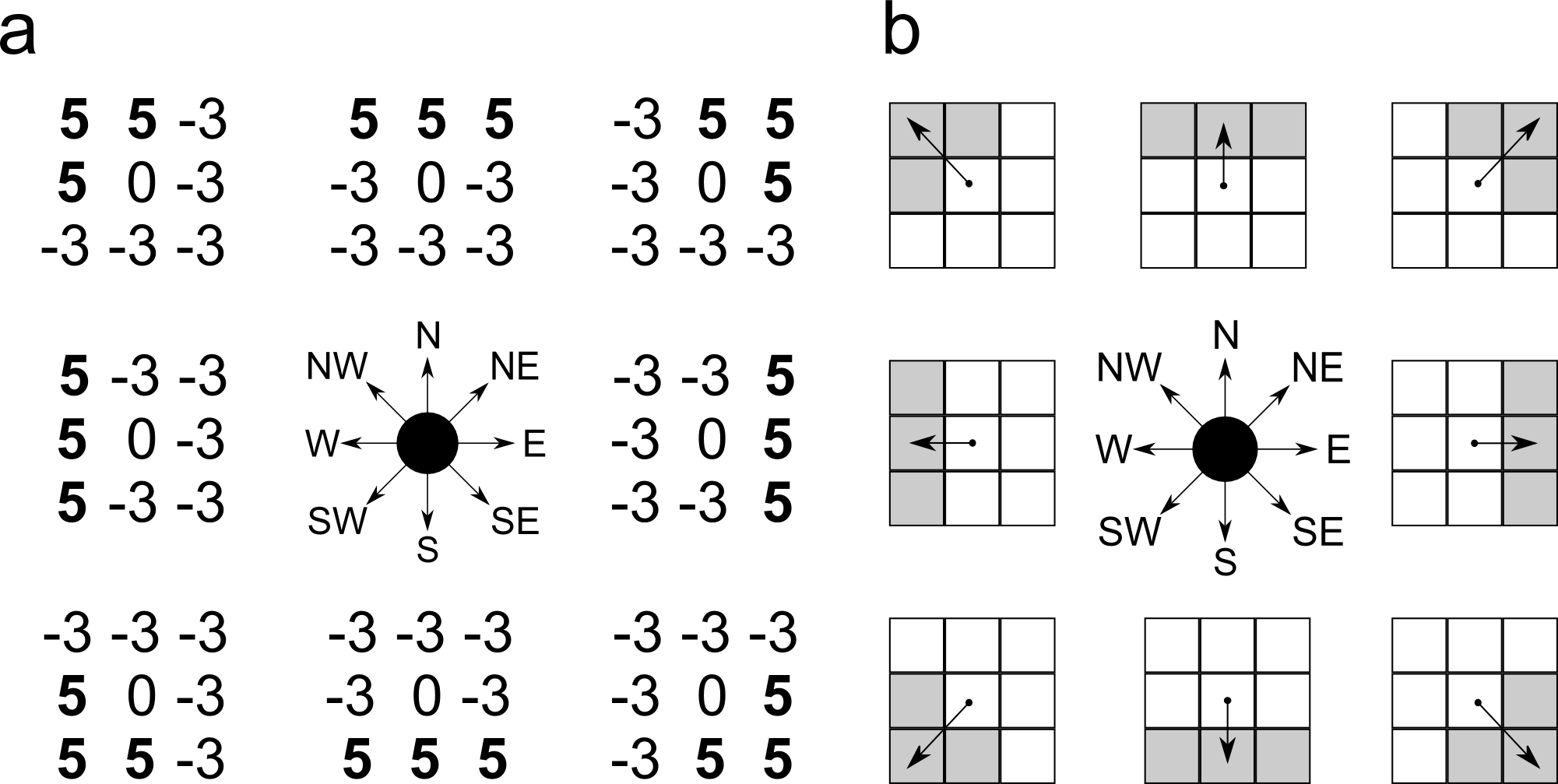

Supplement: Supplementary file 2 — Figure S2. Kirsch kernels for gradient direction computation. (A) Kirsch kernels employed for gradient direction determination. (B) Gradient directions (black arrows) were associated with their closest directions (grey-filled squares): the pixel in the direction of the gradient, and the two pixels in the two adjacent directions. [file bit0111-0504-SD2.tif]

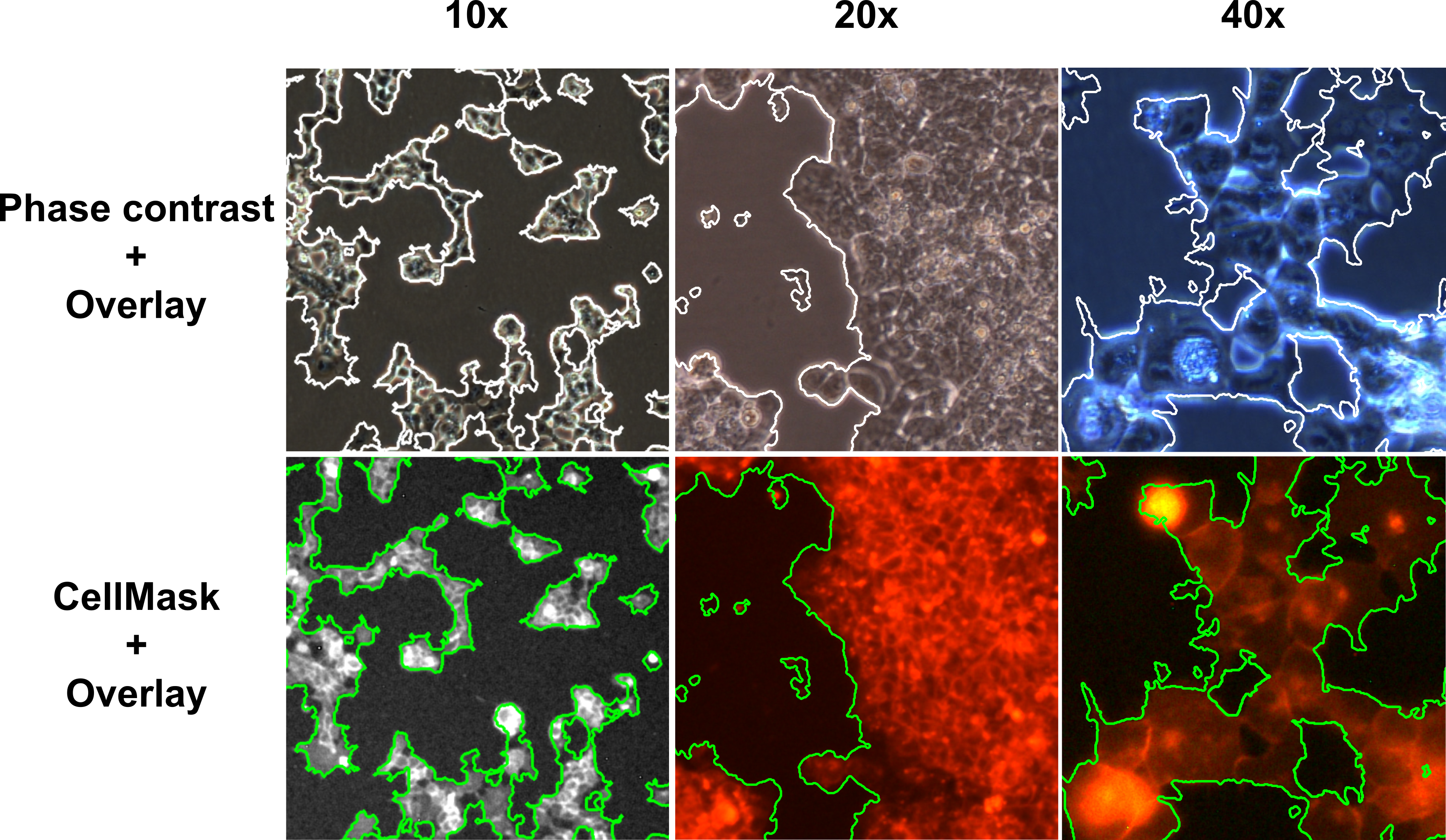

Supplement: Supplementary file 3 — Figure S3. Comparison of segmentation outcome using PHANTAST with a cell membrane fluorescent marker. The top row contains PCM images at three different magnifications overlaid with the segmentation result (white). The bottom row shows the fluorescence image of the cell membrane marker (CellMask) with the same overlay (green). [file bit0111-0504-SD3.tif]

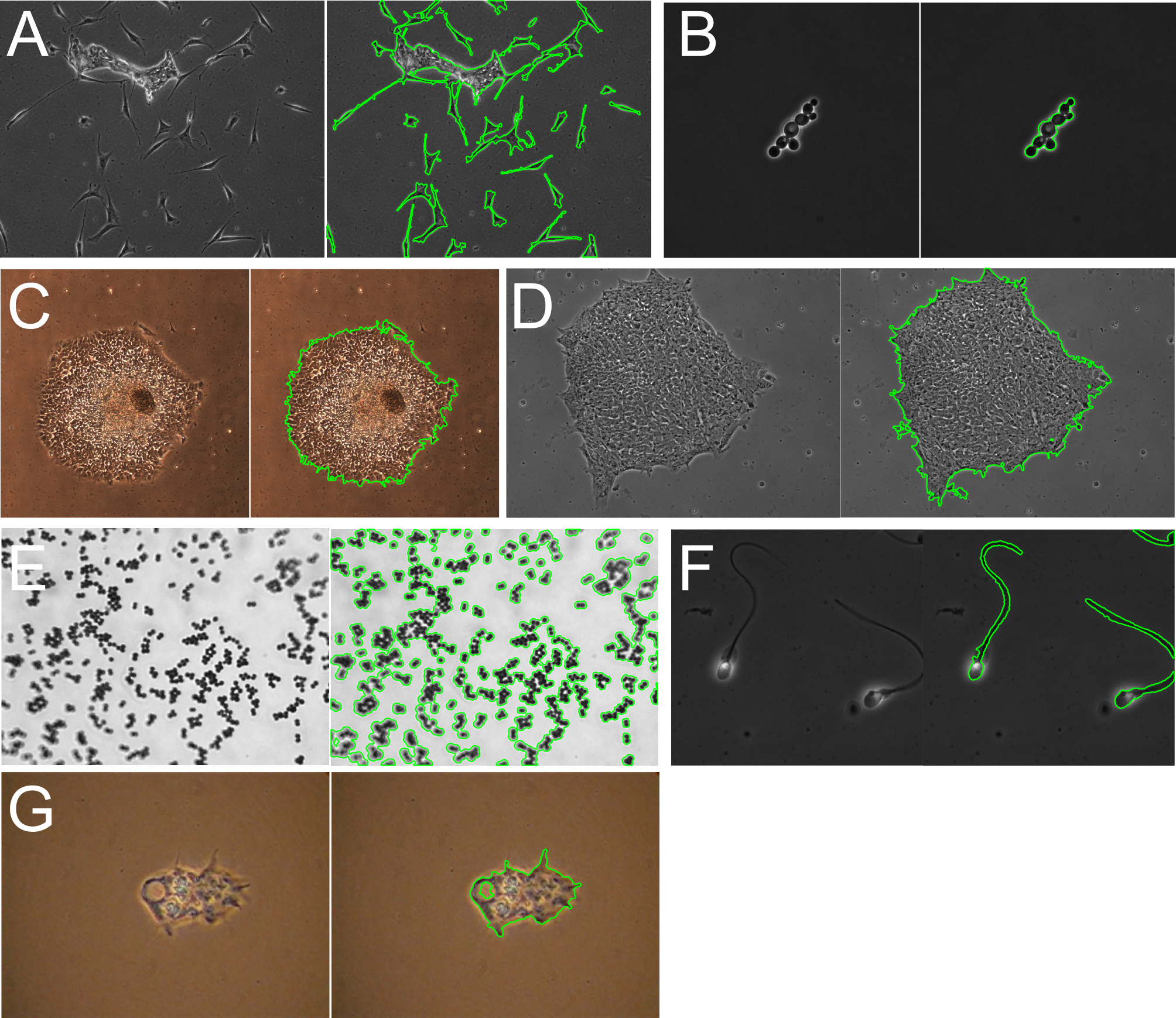

Supplement: Supplementary file 4 — Figure S4. Segmentation results for a wide range of cell types. For each set, the first image is the raw PCM image and the second is the same raw imaged overlaid with the segmentation result in green. (A) NIH/3T3, adapted from Cell: An Image Library (John Elliot, CIL 7883). (B) Saccharomyces cerevisiae, adapted from Cell: An Image Library (David Ball, Jean Peccoud, CIL 39626). (C) Human induced pluripotent stem cells cultured on Matrigel, courtesy of Zuming Tang (University College London, Biochemical Engineering). (D) Human embryonic stem cell colony on Matrigel, adapted from Cell: An Image Library (Sabrina Lin, Prue Talbot, CIL 12626). (E) Pichia pastoris, courtesy of Dr Rochelle Aw (Imperial College London, Division of Molecular Biosciences). (F) Human sperm, adapted from Cell: An Image Library (Michael Crammer, CIL 34524). (G) Acanthamoeba castellani, adapted from Cell: An Image Library (Thelma Dunnebacke, CIL 19326). [file bit0111-0504-SD4.tif]

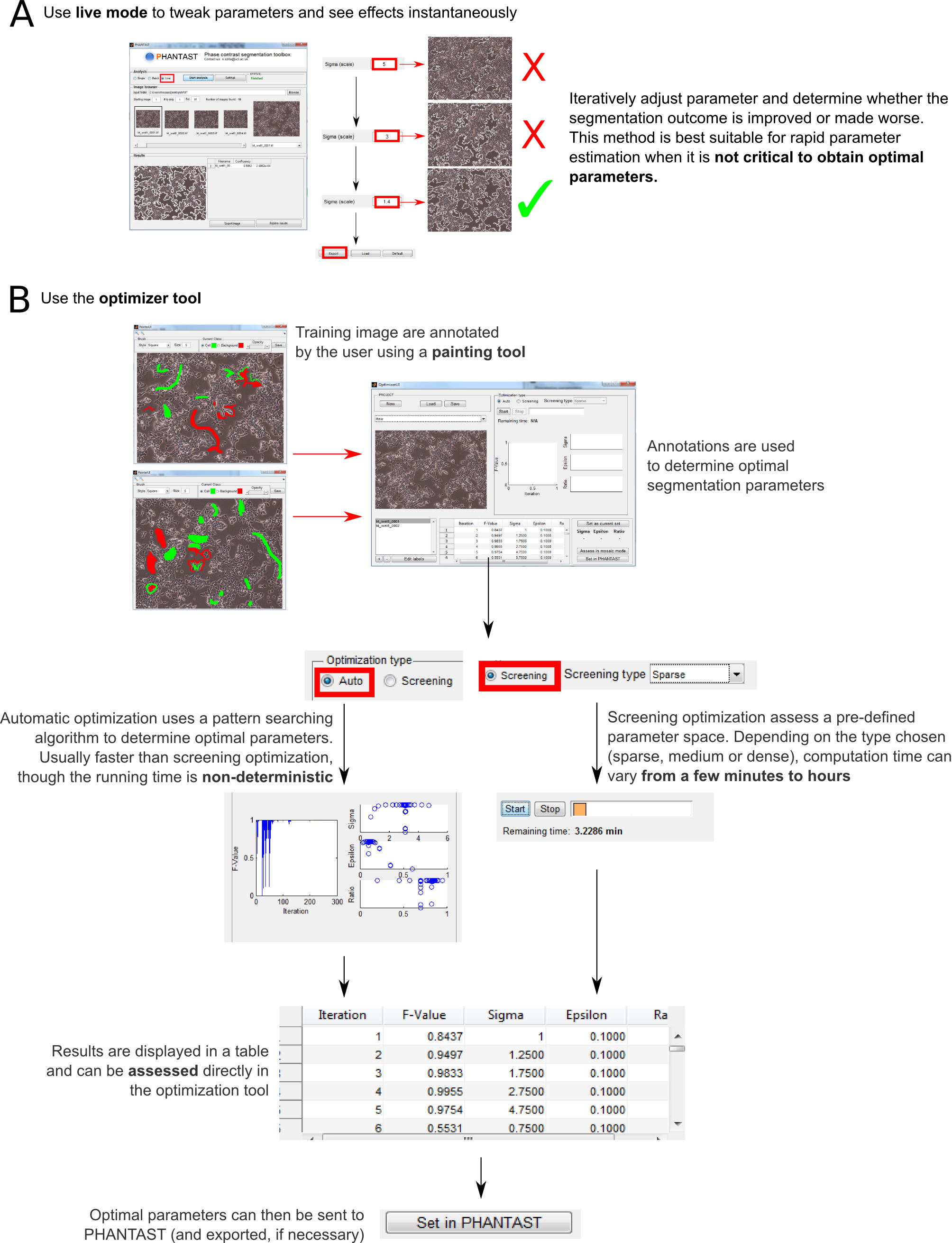

Supplement: Supplementary file 5 — Figure S5. Parameter optimization protocols using (A) the live mode of PHANTAST or (B) the optimization tool. [file bit0111-0504-SD5.tif]

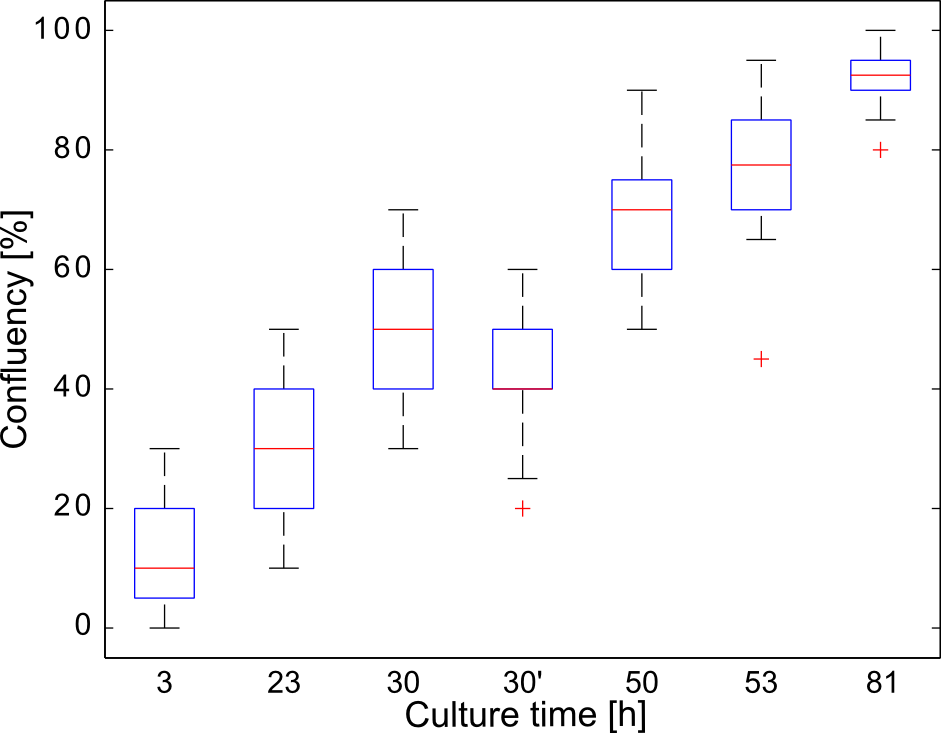

Supplement: Supplementary file 6 — Figure S6. Confluency estimations by 14 experienced human experts based on seven sets of six phase contrast microscopy (PCM) images acquired during mESC expansion. Each researcher was shown seven sets of PCM images. Each set contained six images acquired at random locations within a single well. Each individual was asked to estimate the confluency for each set based on the six images in the set. Six of the seven sets were taken at different time points to represent stages of the expansion process (from shortly after cell seeding to near confluency). A seventh set (30') contained the same images than the ones from the 30 h time point, but its images had all been rotated by 90°, mirrored, and were displayed in a different arrangement. Thus, effectively, two sets of images existed which had identical confluency (images taken at 30 h into the culture). To avoid a bias when estimating the confluency, the sets were not presented in chronological order. To ensure that each individual was faced with the same task, the seven sets were presented in the same order to all researchers. For each box, the central mark is the median, the edges are the 25th and 75th percentiles and the whiskers extend to the most extreme data points (not including outliers). The “+” markers represent outliers. These results highlight the high variability of confluency estimations made by human operators. [file bit0111-0504-SD6.tif]

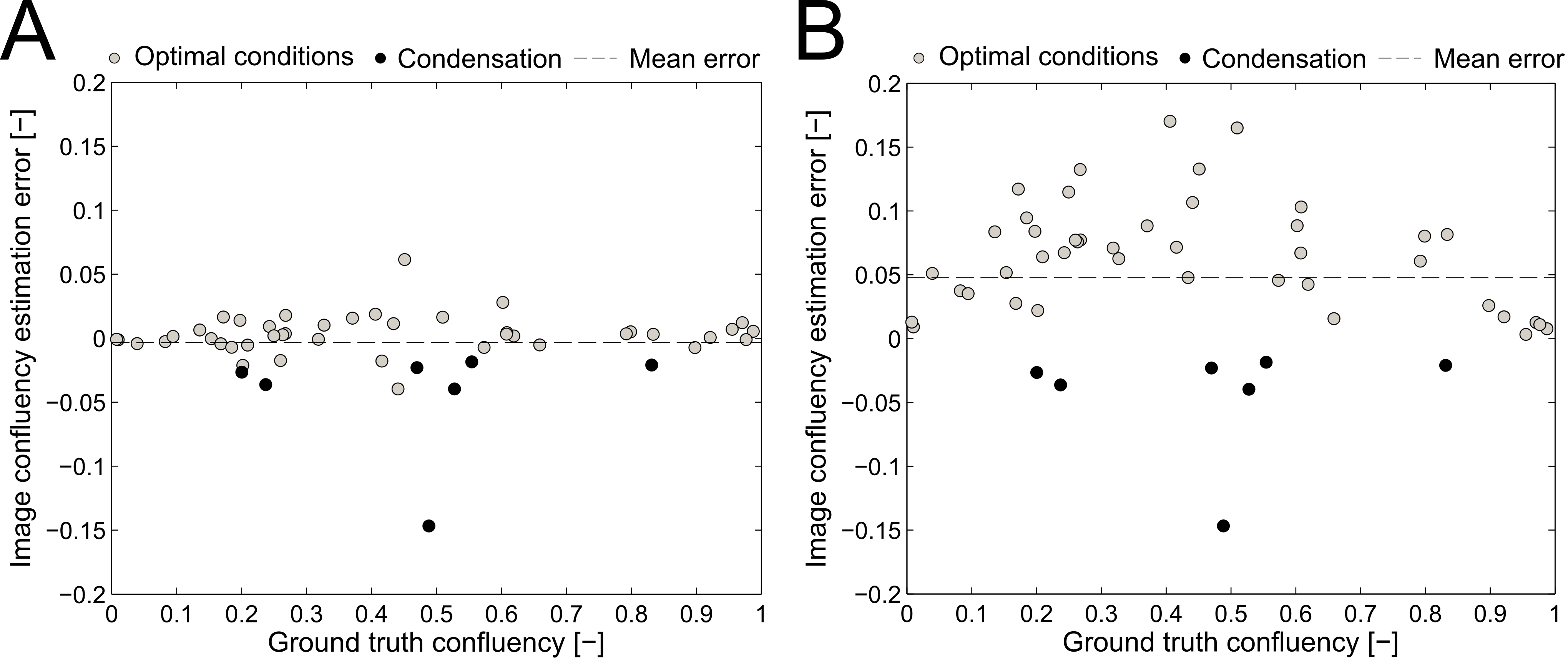

Supplement: Supplementary file 7 — Figure S7. Confluency estimation performance for single images assessed using 50 mESC images. (A) With halo correction: The mean signed difference (bias) for 50 confluency estimates was −0.35% with a 95% confidence interval (CI) of [−1.12%, 0.42%]. The root mean square error (RMSE) of the estimates was 2.69% and the precision, as determined from the RMSE and the bias, was 2.67%. (B) Without halo correction: The mean signed difference (bias) for 50 confluency estimates was 4.78% with a 95% confidence interval (CI) of [2.72%, 6.82%]. The RMSE of the estimates was 8.59% and the precision, as determined from the RMSE and the bias, was 7.14%. [file bit0111-0504-SD7.tif]

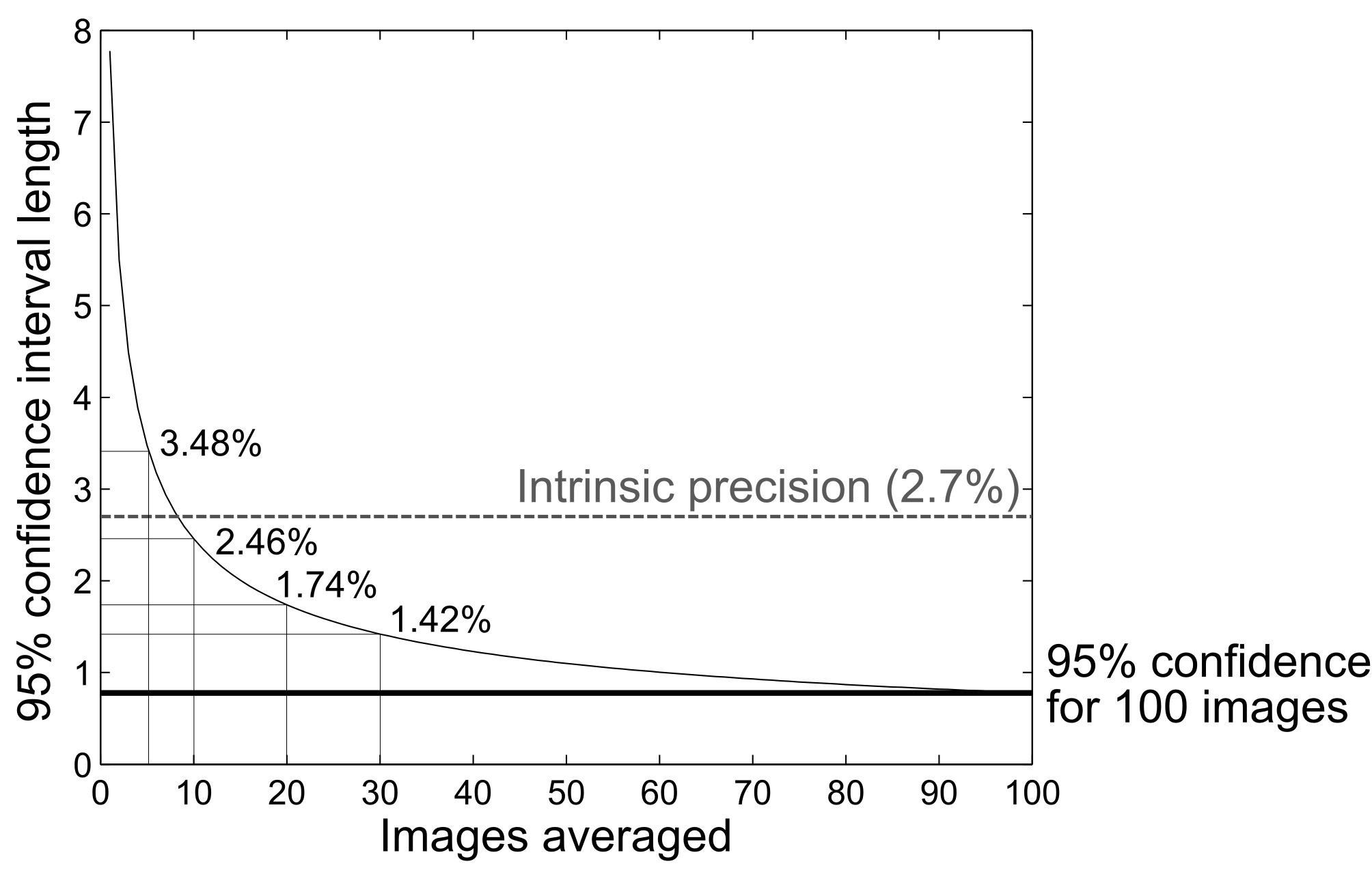

Supplement: Supplementary file 8 — Figure S8. Estimation of the error due to finite sample size. One Hundred images of a mESC culture were acquired and processed. The mean confluency and the corresponding 95% confidence interval were computed. The standard deviation obtained using 100 images can be used to compute the confidence interval length for various sample size as follow: 1.96 * σ/√n where σ is the standard deviation for 100 images and n is the sample size. For comparison purposes only, the intrinsic precision of the algorithm is plotted (dashed lined) in the same graph. [file bit0111-0504-SD8.tif]

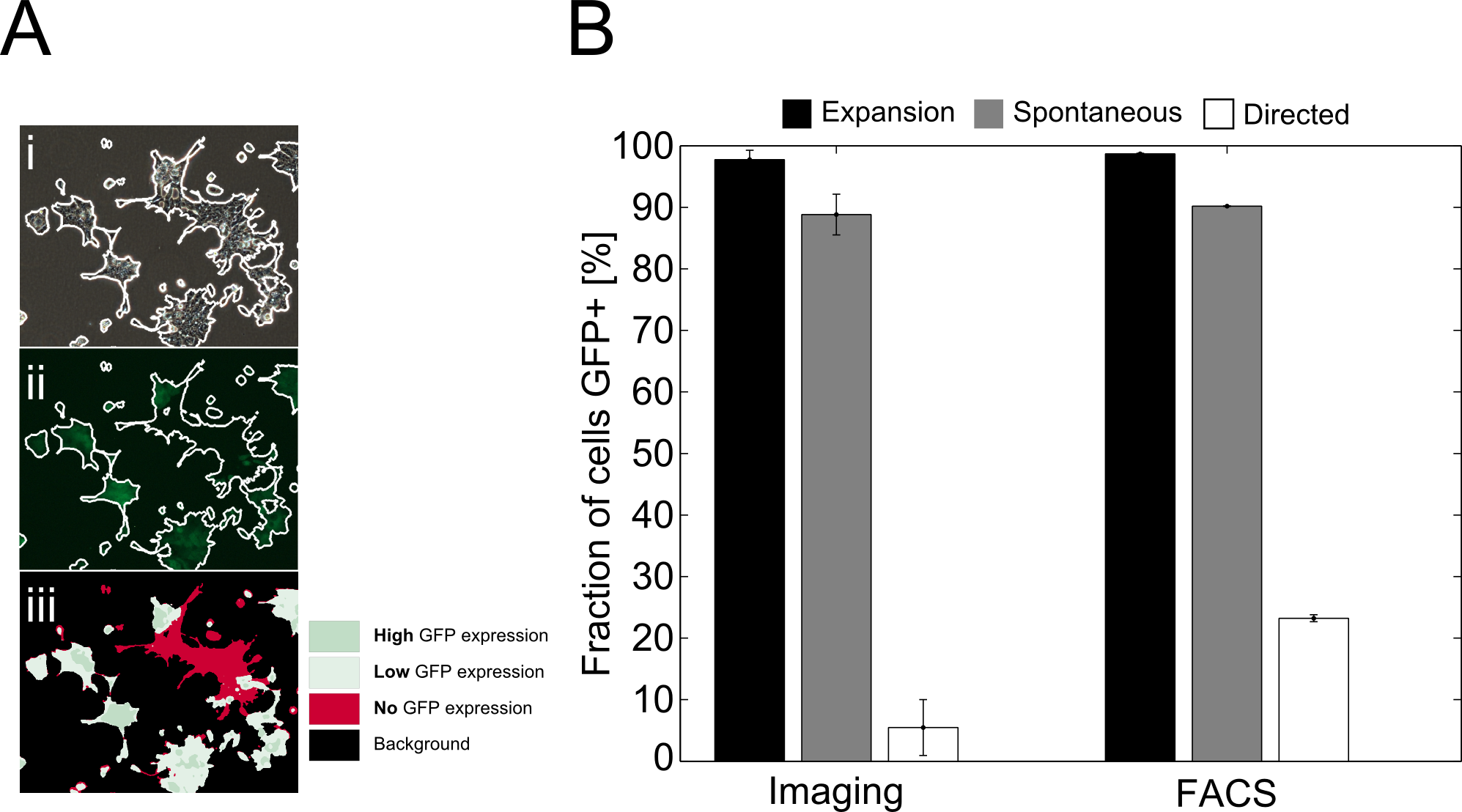

Supplement: Supplementary file 9 — Figure S9. Determination of spatial and temporal expression pattern of a fluorescent reporter. (A) Combination of a segmented phase contrast image (i) with a fluorescence image, here with the segmentation overlaid in white (ii) in order to generate an abstract representation showing expressing regions (light and dark green for low and high expression, respectively), regions with cells that do no express the reporter (red) and background with no cells (black). (B) Comparison of the results of the determination of GFP+ cells using the imaging method described in A with flow cytometry. [file bit0111-0504-SD9.tif]

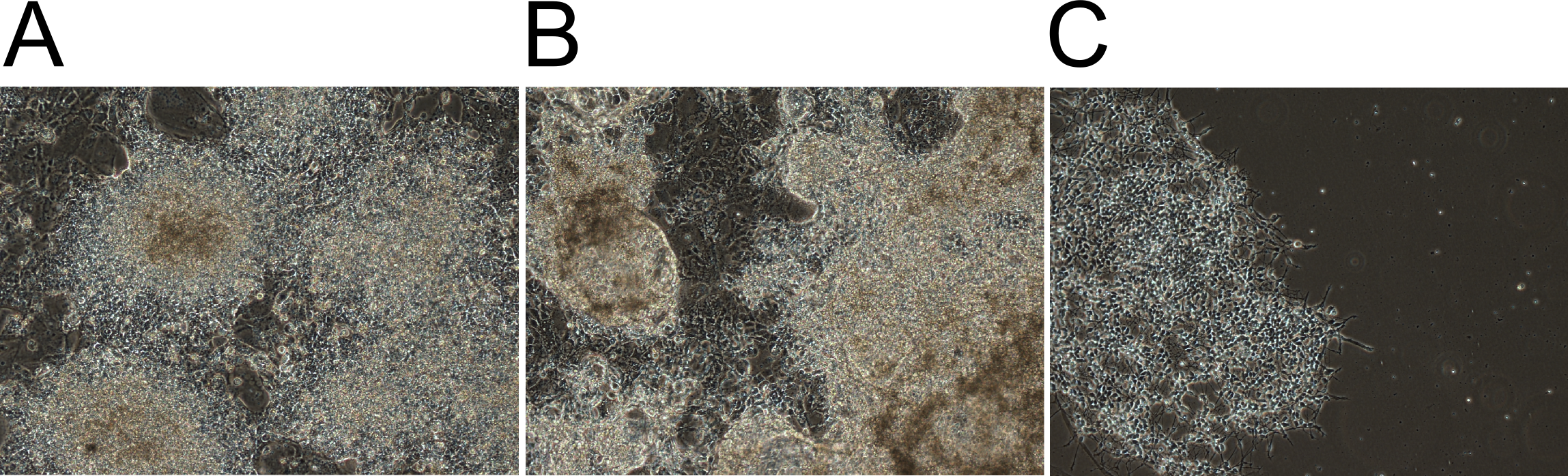

Supplement: Supplementary file 10 — Figure S10. Phase contrast images of mESC cells after 14 days of culture in (A) expansion medium with LIF, (B) spontaneous differentiation medium without LIF, and (C) directed differentiation medium. [file bit0111-0504-SD10.tif]

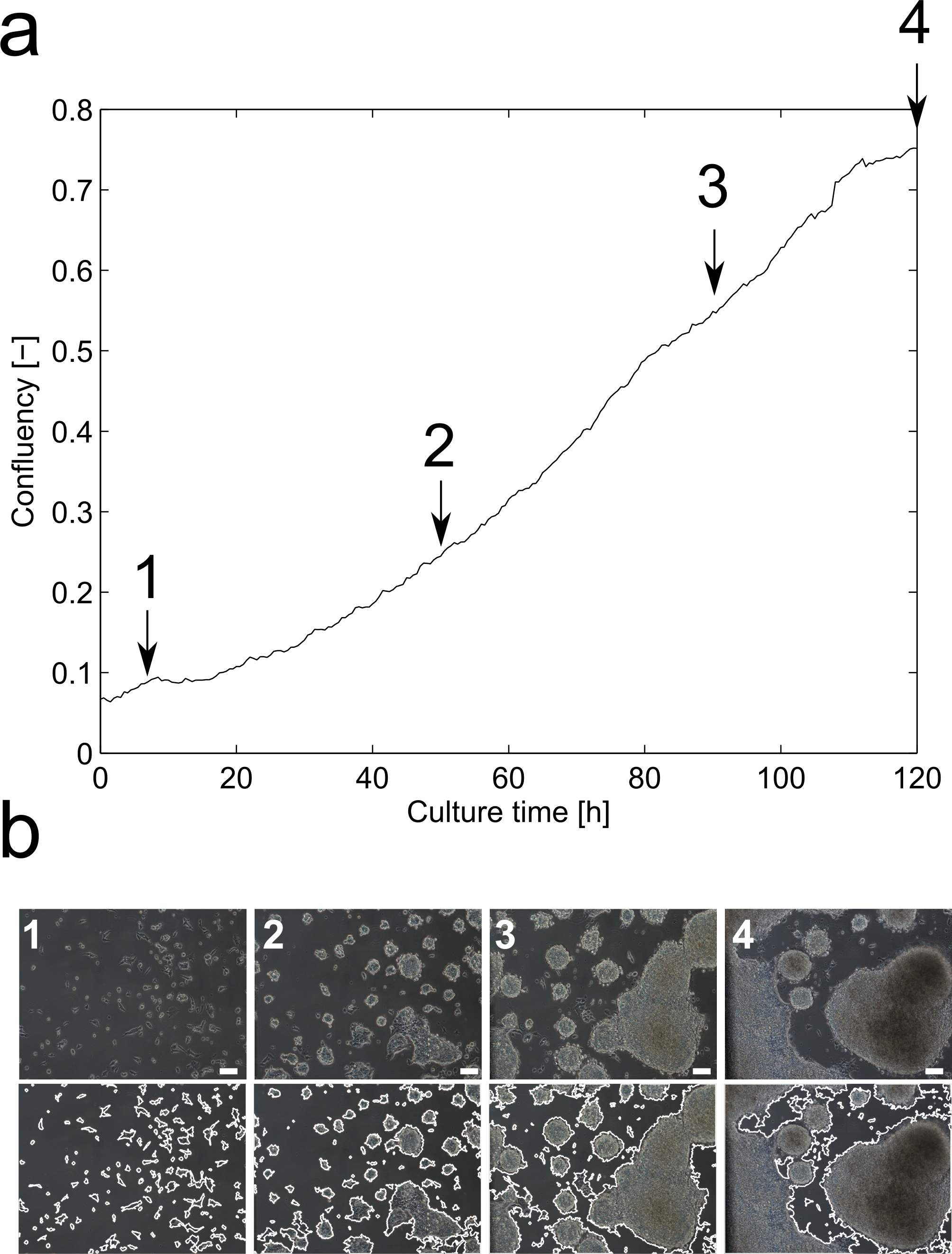

Supplement: Supplementary file 11 — Figure S11. Mouse embryonic stem cell growth monitoring in a microfabricated bioreactor. (a) Confluency measurements for a single field of view imaged over 5 days. An image was automatically acquired every 30 min. (b) Phase contrast images acquired at the time points indicated in a (first row) and images overlaid with the contours detected by PHANTAST (second row). Scale bars are 100 µm. [file bit0111-0504-SD11.tif]
