# Supplementary material for: Automated Method for the Rapid and Precise Estimation of Adherent Cell Culture Characteristics from Phase Contrast Microscopy Images
Source: Biotechnol Bioeng. 2013 Oct 5;111(3):504–17. doi: 10.1002/bit.25115 (PMC4260842; doi:10.1002/bit.25115)
Supplement: Supplementary file 12 — Table SI. Optimised parameters for the segmentation algorithm. The investigated range refers to the range of values screened during the optimization process. [file bit0111-0504-SD12.doc]

**Supplementary Table 1**. Optimised parameters for the segmentation algorithm. The investigated range refers to the range of values screened during the optimisation process.

| **Step** | **Symbol** | **Name** | **Value** | **Investigated range** |
| --- | --- | --- | --- | --- |
| **Local contrast** | σ | Local contrast filter scale | 1.4 | [0.1, 2] |
|  | ε | Global intensity threshold | 0.03 | [0.01, 0.2] |
| **Halo correction** | F_max_ | Max fill area | 25 | [0, 800] |
|  | R_max_ | Small object removal threshold | 325 | [0, 100] |
|  | A_ratio_ | Portion of an object that can be removed | 0.3 | [0, 1] |
