# Supplementary material for: Automated Method for the Rapid and Precise Estimation of Adherent Cell Culture Characteristics from Phase Contrast Microscopy Images
Source: Biotechnol Bioeng. 2013 Oct 5;111(3):504–17. doi: 10.1002/bit.25115 (PMC4260842; doi:10.1002/bit.25115)
Supplement: Supplementary file 13 — Table SII. Number of images required to cover the whole culture area of various commonly used culture vessels. These calculations are based on a field of view of approximately 1.2 mm2. [file bit0111-0504-SD13.doc]

**Supplementary Table 2**. Number of images required to cover the whole culture area of various commonly used culture vessels. These calculations are based on a field of view of approximately 1.2 mm^2^.

| **Vessel** | **Approximate growth area [cm^2^]** | **10× images to cover entire growth area** |
| --- | --- | --- |
| 96-well | 0.32 | 27 |
| 48-well | 0.95 | 81 |
| 24-well | 1.9 | 161 |
| 12-well | 3.8 | 322 |
| 6-well | 9.6 | 814 |
| T-25 | 25 | 2121 |
| T-75 | 75 | 6362 |
| T-125 | 125 | 10604 |
