# Supplementary material for: Automated Method for the Rapid and Precise Estimation of Adherent Cell Culture Characteristics from Phase Contrast Microscopy Images
Source: Biotechnol Bioeng. 2013 Oct 5;111(3):504–17. doi: 10.1002/bit.25115 (PMC4260842; doi:10.1002/bit.25115)
Supplement: Supplementary file 14 — Table SIII. Cross-validation of the cell density estimation method based on three cultures in 6 well-plates that spanned the whole range of possible confluencies. A linear regression between packing-corrected confluency (PCC) and cell density after counting was done for one culture and used to predict the cell density of the other 2. For comparison purposes, the same validation was done by using confluency instead of PCC. [file bit0111-0504-SD14.doc]

**Supplementary Table 3**. Cross-validation of the cell density estimation method based on 3 cultures in 6 well-plates that spanned the whole range of possible confluencies. A linear regression between packing-corrected confluency (PCC) and cell density after counting was done for one culture and used to predict the cell density of the other 2. For comparison purposes, the same validation was done by using confluency instead of PCC.

|  | **Packing-corrected confluency** | | **Confluency** | |
| --- | --- | --- | --- | --- |
| **Metric** | **Mean** | **Standard deviation** | **Mean** | **Standard deviation** |
| RMSE [cells] | 4.7*10^4^ | 8.1*10^3^ | 9.8*10^4^ | 1*10^4^ |
| nRMSE [%] | 10.2 | 2.1% | 21.5 | 1.6% |
| Bias [cells] | 717.9 | 8.8*10^3^ | 302 | 9.6*10^3^ |
| Mean relative error [%] | 5.9% | 6.2% | 25.7% | 2% |
| Adjusted r-squared [-] | 0.98 | 0.02 | 0.88 | 0.04 |
